# Robust joint score tests in the application of DNA methylation data analysis

**DOI:** 10.1186/s12859-018-2185-3

**Published:** 2018-05-18

**Authors:** Xuan Li, Yuejiao Fu, Xiaogang Wang, Weiliang Qiu

**Affiliations:** 1 Department of Mathematics and Statistics, York University, 4700 Keele Street, Toronto, M3J1P3 Canada; 2 Channing Division of Network Medicine, Brigham and Women’s Hospital, Harvard Medical School, 181 Longwood Avenue, Boston, 02115 USA

**Keywords:** Methylation data, Joint score tests, Variability

## Abstract

**Background:**

Recently differential variability has been showed to be valuable in evaluating the association of DNA methylation to the risks of complex human diseases. The statistical tests based on both differential methylation level and differential variability can be more powerful than those based only on differential methylation level. Anh and Wang (2013) proposed a joint score test (AW) to simultaneously detect for differential methylation and differential variability. However, AW’s method seems to be quite conservative and has not been fully compared with existing joint tests.

**Results:**

We proposed three improved joint score tests, namely iAW.Lev, iAW.BF, and iAW.TM, and have made extensive comparisons with the joint likelihood ratio test (jointLRT), the Kolmogorov-Smirnov (KS) test, and the AW test. Systematic simulation studies showed that: 1) the three improved tests performed better (i.e., having larger power, while keeping nominal Type I error rates) than the other three tests for data with outliers and having different variances between cases and controls; 2) for data from normal distributions, the three improved tests had slightly lower power than jointLRT and AW. The analyses of two Illumina HumanMethylation27 data sets GSE37020 and GSE20080 and one Illumina Infinium MethylationEPIC data set GSE107080 demonstrated that three improved tests had higher true validation rates than those from jointLRT, KS, and AW.

**Conclusions:**

The three proposed joint score tests are robust against the violation of normality assumption and presence of outlying observations in comparison with other three existing tests. Among the three proposed tests, iAW.BF seems to be the most robust and effective one for all simulated scenarios and also in real data analyses.

**Electronic supplementary material:**

The online version of this article (10.1186/s12859-018-2185-3) contains supplementary material, which is available to authorized users.

## Background

DNA methylation is an epigenetic mechanism that regulates gene expression without changing genetic codes. Usually, DNA methylation inhibits the expression of its nearby gene by adding a methyl group to the fifth carbon atom of a cytosine ring. Since it is a reversible biological process, DNA methylation is now considered as a potential therapeutic target in cancer treatment due to its ability to inhibit the expression of oncogenes which can transform a cell into a tumor cell in certain circumstances.

One major goal in the analysis of methylation data is to identify disease-associated CpG sites. Many analyses in the past have been focused on the difference of average or mean methylation levels between the disease and the control group. However, it has not been a common practice in the classical statistical analysis to test a hypothesis of equal variances since the difference of population means between the disease and control group is normally the inferential interest. Recently, some evidence suggests that the epigenetic variation is also a very important intrinsic characteristic associated with certain diseases [1–6]. These papers in DNA methylation analyses showed that differentially variable DNA methylation marks are biologically relevant to the disease of interest since the genes regulated by these marks are enriched in the biological pathways that have been found important to the disease of interest.

Although there are more than 50 statistical tests for equal variance [7], several new methods have been proposed especially for the analysis of DNA methylation data [2, 8]. We recently compared these new methods [4] and proposed three improved equal variance tests based on the score test of logistic regression [6]. Since both mean and variance are biologically meaningful in DNA methylation analysis, it is logical to simultaneously test for equal mean and equal variance. The joint likelihood ratio test (jointLRT) and the two-sample Kolmogorov-Smirnov (KS) test are two traditional methods for this task. Recently Anh and Wang (2013) [8] proposed a new joint test based on logistic regression (AW), which is essentially a quadratic form of a vector of two tests. One of them is to test for equal means; the other is to test for equal variances. However, they did not provide the asymptotic distribution of their test statistic nor the comparison of their joint test with jointLRT or KS that are the benchmark tests in the statistical literature.

In this article, we derived the asymptotic distribution of the AW joint test statistic and made comprehensive comparisons between AW, jointLRT and KS tests. Although a normal distribution is usually assumed for methylation data, the violation of normality assumption and presence of outlying points can often be observed in the analysis of real data. Bi-modal distributions are also encountered frequently in practice. To improve the power and robustness of the AW joint test, we proposed three tests based on absolute deviation from mean (iAW.Lev), median (iAW.BF) and trimmed mean (iAW.TM) respectively.

Results from our simulation studies suggest that the three improved tests are robust in skewed distributions and (unimodal) distributions with outliers. Among the three improved tests, iAW.BF is the most robust in mixtures of two normal distributions and also in other scenarios. Results of real data analyses presented that iAW.BF and iAW.TM performed significantly better than AW, jointLRT, and KS. Although iAW.Lev works well in the simulation setting, it does not seem to be very stable in terms of the proportion of true validation in real data analyses.

## Methods

### Justification for Ahn and Wang’s joint score test

Ahn and Wang (2013) [8] proposed a joint score test to detect methylation marks relevant to a disease. Their approach tests for homogeneity of means and variances simultaneously. Since Ahn and Wang (2013) [8] did not provide a detailed theoretical proof for the asymptotic distribution of this joint score test, we now fill this gap in theory.

Let *X*_*i*_ and *Y*_*i*_ denote the methylation value and the corresponding disease status of subject *i*, where *i*=1,2,…,*n*, with *n*=*n*_0_+*n*_1_, *n*_0_ is the number of the non-diseased subjects (controls, *Y*_*i*_=0) and *n*_1_ is the number of the diseased subjects (cases, *Y*_*i*_=1). To detect methylation loci that are relevant to a disease based on means and variances, the corresponding hypothesis is formulated as *H*_0_:*μ*_0_=*μ*_1_ and $\sigma _{0}^{2}=\sigma _{1}^{2}$ versus *H*_1_:*μ*_0_≠*μ*_1_ or $\sigma _{0}^{2} \neq \sigma _{1}^{2}$, in which *μ*_0_ and *μ*_1_ are means of methylation levels for controls and cases, respectively, and $\sigma _{0}^{2}$ and $\sigma _{1}^{2}$ are the corresponding variances.

Instead of directly testing the above hypothesis, Ahn and Wang (2013) [8] proposed to test *H*0′:*β*_1_=*β*_2_=0 versus *Ha*′:*β*_1_≠0 or *β*_2_≠0, where *β*_1_ and *β*_2_ are the regression coefficients of the following logistic regression:

1 $$\begin{array}{@{}rcl@{}} logit\left[Pr(Y_{i}=1|x_{i},z_{i})\right]=\beta_{0}+\beta_{1} x_{i}+\beta_{2} z_{i}, \end{array} $$

and *z*_*i*_ is the within-group squared deviation for subject *i*, which is defined as

2 $$ z_{i}=\left\{\begin{array}{ll} \left(x_{i}-\bar{x}_{1}\right)^{2}, \text{if } Y_{i}=1,\\ \left(x_{i}-\bar{x}_{0}\right)^{2}, \text{if } Y_{i}=0, \end{array}\right.  $$

and $\bar {x}_{1}=\sum _{i=1}^{n}x_{i}I\left [y_{i}=1\right ]/n_{1}$ and $\bar {x}_{0}=\sum _{i=1}^{n}x_{i}I \left [y_{i}=0\right ]/n_{0}$ are the sample means for cases and controls.

The AW test statistic $T=\mathbf {U}^{T}\widehat {\mathbf {\Sigma }}^{-1}\mathbf {U}$ is a quadratic form of two score statistics *U*_1_ and *U*_2_ for the above logistic regression, where **U**=(*U*_1_,*U*_2_)^*T*^,

3 $$ \begin{aligned} U_{1}=&\sum_{i=1}^{n}x_{i}\left(y_{i} - \bar{y}\right),\\ U_{2}=&\sum_{i=1}^{n}z_{i}\left(y_{i} - \bar{y}\right), \end{aligned}  $$

and $\widehat {\mathbf {\Sigma }}$ is the estimate of the covariance matrix *Cov*(**U**).

Under *H*0′, the estimated covariance matrix $\widehat {\mathbf {\Sigma }}$ has the following form:

$$\widehat{\mathbf{\Sigma}}=n\bar{y}\left(1-\bar{y}\right) \left(\begin{array}{cc} \hat{\sigma}_{x}^{2}&\hat{\sigma}_{xz}\\ \hat{\sigma}_{xz}&\hat{\sigma}_{z}^{2} \end{array} \right), $$

where $\hat {\sigma }_{x}^{2}=\sum _{i=1}^{n}(x_{i}-\bar {x})^{2}/n$ and $\hat {\sigma }_{z}^{2}=\sum _{i=1}^{n}(z_{i}-\bar {z})^{2}/n$ are the sample variances for *x*_*i*_ and *z*_*i*_, and $\hat {\sigma }_{xz}=\sum _{i=1}^{n} (x_{i}-\bar {x})(z_{i}-\bar {z})/n$ is the sample covariance between *x*_*i*_ and *z*_*i*_.

Note that in logistic regression (1), the random variables are *y*_*i*_, while *x*_*i*_ and *z*_*i*_ are fixed (i.e., non-random). Hence, the (asymptotic) distributions of the *U*_1_, *U*_2_, and *T* do not depend on the distributions of *x*_*i*_ and *z*_*i*_. In this sense, we can say that the AW test statistic *T* is theoretically robust against the violation of the normality assumption for the predictors *x*_*i*_ and *z*_*i*_.

Dobson (1990) [9] showed that $\mathbf {U} \stackrel {H_{0}'}{\rightarrow } N(\mathbf {0},Cov(\mathbf {U}))$. When the sample size is large, the asymptotic distribution of *T* is $\chi _{2}^{2}$ under *H*0′, based on the Law of Large Numbers and the relationship between the multivariate normal distribution and the chi-squared distribution. Ascribed to limited space, the complete proof is included in the Additional file 1.

#### Three improved joint score tests

Since the within-group squared deviation in (2) might not be very robust, we propose three improved joint score tests.

In the first improved joint score test (denoted as iAW.Lev), we replace the within-group squared deviation by within-group absolute deviation [10]:

4 $$\begin{array}{@{}rcl@{}} z_{i}^{*}= \left\{ \begin{array}{ll} |x_{i}-\bar{x}_{1}|, \text{if } Y_{i}=1,\\ |x_{i}-\bar{x}_{0}|, \text{if } Y_{i}=0. \end{array} \right. \end{array} $$

For the logistic regression $logit \left [Pr \left (Y_{i}=1 |x_{i},z_{i}^{*}\right)\right ]=\beta _{0}^{*}+\beta _{1}^{*}x_{i}+\beta _{2}^{*}z_{i}^{*}$, under the null hypothesis $H_{0}^{*}$: $\beta _{1}^{*}=\beta _{2}^{*}=0$, the joint score test statistic *T*^*Lev*^ is asymptotically chi-squared distributed with two degrees of freedom:

$$\begin{array}{@{}rcl@{}} T^{Lev}=\left(\mathbf{U}^{Lev}\right)^{T}\left(\widehat{\mathbf{\Sigma}}^{Lev}\right)^{-1}\mathbf{U}^{Lev} \stackrel{H_{0}^{*}}{\rightarrow} \chi^{2}_{2}, \end{array} $$

where $\mathbf {U}^{Lev}=\left (U_{1}, U_{2}^{*}\right)^{T}$, $U_{2}^{*}=\sum _{i=1}^{n}z_{i}^{*}\left (y_{i}-\bar {y}\right)$,

$$\widehat{\mathbf{\Sigma}}^{Lev}=n\bar{y}\left(1-\bar{y}\right) \left(\begin{array}{cc} \hat{\sigma}_{x}^{2}&\hat{\sigma}_{xz^{*}}\\ \hat{\sigma}_{xz^{*}}&\hat{\sigma}_{z^{*}}^{2} \end{array} \right), $$

where $\hat {\sigma }_{z^{*}}^{2}$ is the sample variance for $z_{i}^{*}$, and $\hat {\sigma }_{xz^{*}}$ is the sample covariance between *x*_*i*_ and $z_{i}^{*}$. Note that the proposed improved joint test is different from Levene’s test [10] in that Levene’s test regards $z_{i}^{*}$ as random and uses ANOVA, while the proposed improved joint test regards $z_{i}^{*}$ as fixed (i.e., non-random) and uses a logistic regression framework.

In the second improved joint score test, we replace the sample means in the *T*^*Lev*^ by sample medians [11]:

5 $$\begin{array}{@{}rcl@{}} z_{i}^{BF}= \left\{ \begin{array}{ll} |x_{i}-\tilde{x}_{1}|, \text{if } Y_{i}=1,\\ |x_{i}-\tilde{x}_{0}|, \text{if } Y_{i}=0, \end{array} \right. \end{array} $$

where $\tilde {x}_{1}$ and $\tilde {x}_{0}$ are the sample medians for cases and controls respectively. Under the null hypothesis $H_{0}^{BF}: \beta _{0}^{BF}=\beta _{1}^{BF}=0$, the joint score test statistic *T*^*BF*^ follows asymptotically the chi-squared distribution with two degrees of freedom:

$$\begin{array}{@{}rcl@{}} T^{BF}=\left(\mathbf{U}^{BF}\right)^{T}\left(\widehat{\mathbf{\Sigma}}^{BF}\right)^{-1}\mathbf{U}^{BF}\stackrel{H_{0}^{BF}}{\rightarrow} \chi^{2}_{2}, \end{array} $$

where $\mathbf {U}^{BF}=\left (U_{1}, U_{2}^{BF}\right)^{T}$, $U_{2}^{BF}=\sum _{i=1}^{n}z_{i}^{BF}\left (y_{i}-\bar {y}\right)$,

$$\widehat{\mathbf{\Sigma}}^{BF}=n\bar{y}\left(1-\bar{y}\right) \left(\begin{array}{cc} \hat{\sigma}_{x}^{2}&\hat{\sigma}_{xz^{BF}}\\ \hat{\sigma}_{xz^{BF}}&\hat{\sigma}_{z^{BF}}^{2} \end{array} \right), $$

where $\hat {\sigma }_{z^{BF}}^{2}$ is the sample variance for $z_{i}^{BF}$, and $\hat {\sigma }_{xz^{BF}}$ is the sample covariance between *x*_*i*_ and $z_{i}^{BF}$.

In the third improved joint score test, we replace the sample means in the *T*^*Lev*^ by trimmed sample means [11]:

6 $$\begin{array}{@{}rcl@{}} z_{i}^{TM}= \left\{ \begin{array}{ll} |x_{i}-\check{x}_{1}|, \text{if } Y_{i}=1,\\ |x_{i}-\check{x}_{0}|, \text{if } Y_{i}=0, \end{array} \right. \end{array} $$

where $\check {x}_{1}$ and $\check {x}_{0}$ are the 25% trimmed sample means for cases and controls respectively. The 25% trimmed mean for a sample is the sample mean after trimming 25% lowest values and 25% highest values.

For the logistic regression model $logit \left [Pr \left ({\vphantom {z_{i}^{TM}}} Y_{i}=1\right.\right. \left.\left. |x_{i},z_{i}^{TM}\right)\right ] =\beta _{0}^{TM}+\beta _{1}^{TM}x_{i}+\beta _{2}^{TM}z_{i}^{TM}$, under the null hypothesis $H_{0}^{TM}$: $\beta _{1}^{TM}=\beta _{2}^{TM}=0$, the joint score test statistic *T*^*TM*^ is asymptotically chi-squared distributed with two degrees of freedom:

$$\begin{array}{@{}rcl@{}} T^{TM}=\left(\mathbf{U}^{TM}\right)^{T}\left(\widehat{\mathbf{\Sigma}}^{TM}\right)^{-1}\mathbf{U}^{TM} \stackrel{H_{0}^{TM}}{\rightarrow} \chi^{2}_{2}, \end{array} $$

where $\mathbf {U}^{TM}=\left (U_{1}, U_{2}^{TM}\right)^{T}$, $U_{2}^{TM}=\sum _{i=1}^{n}z_{i}^{TM}\left (y_{i}-\bar {y}\right)$,

$$\widehat{\mathbf{\Sigma}}^{TM}=n\bar{y}\left(1-\bar{y}\right) \left(\begin{array}{cc} \hat{\sigma}_{x}^{2}&\hat{\sigma}_{xz^{TM}}\\ \hat{\sigma}_{xz^{TM}}&\hat{\sigma}_{z^{TM}}^{2} \end{array} \right), $$

where $\hat {\sigma }_{z^{TM}}^{2}$ is the sample variance for $z_{i}^{TM}$, and $\hat {\sigma }_{xz^{TM}}$ is the sample covariance between *x*_*i*_ and $z_{i}^{TM}$.

## Results

### Simulation studies

We have conducted comprehensive simulations to compare the performances of the three improved tests with the three existing methods: the joint likelihood ratio test based on the normal distribution (jointLRT) [12, 13], the Kolmogorov-Smirnov test (KS) [14], and Ahn and Wang’s joint score test (AW). We have attained the mathematical expression and the exact distribution of jointLRT test statistics under normal distribution [15]. Due to computational complexity, we used the asymptotic distribution of jointLRT in our simulation studies.

The simulation studies examined the following four aspects and their impacts on these six tests: (1) various sample sizes, (2) the presence of heterogeneity of means and variances, (3) the violation of the normality assumption, and (4) outliers. We considered various sample sizes: (*n*_0_,*n*_1_) =(100, 100), (*n*_0_,*n*_1_) =(50, 50), and (*n*_0_,*n*_1_)=(20, 20). Four parametric models were employed to generate the methylation data: the normal distribution, the Beta distribution, the chi-square distribution, and the mixture of two normal distributions. To evaluate the impact of outliers, we replaced the DNA methylation level of one randomly picked disease subject by *max*{*x*_1,*max*_,(*Q*_3_+3(*Q*_3_−*Q*_1_))}, where *x*_1,*max*_ denotes the maximum DNA methylation level of the diseased samples, and *Q*_1_ and *Q*_3_ are the first and third quartiles respectively.

We computed the empirical Type I error rates and the powers of the six tests under different scenarios: (1) Type I error scenario (eqM & eqV): distributions of non-diseased and diseased samples are the same; (2) Power scenario I (diffM & eqV): distributions of non-diseased and diseased samples are different in means only; (3) Power scenario II (eqM & diffV): distributions of non-diseased and diseased samples are different in variances only; and (4) Power scenario III (diffM & diffV): distributions of non-diseased and diseased samples are different in both means and variances. We conducted 10,000 simulations to estimate Type I error rates for scenario (1). For the remaining 3 scenarios, 5000 simulations are conducted to estimate the power of a test using the corrected cutoff values obtained in scenario (1) so that corrected Type I error rates are approximately equal to the nominal Type I error rates.

Overall, the three improved joint score tests performed better than the other three methods when methylation levels contained outliers and had different variances between diseased and non-diseased samples. Besides, iAW.BF is the most robust in terms of power among all the scenarios. The KS test had conservative empirical Type I error rates and lowest power in many scenarios.

When methylation levels were generated based on normal distributions without outliers, all tests had the empirical Type I error rates close to the nominal levels, except for KS (Table 1). For Power Scenarios I, II and III, three improved joint score tests had similar performances, but slightly lower power for jointLRT and AW. When methylation values were from normal distributions with an outlier, the three improved joint score tests can keep empirical Type I error rates well at all nominal levels. Whereas the empirical Type I error rates of jointLRT were inflated at all nominal levels, AW and KS had very conservative empirical Type I error rates at all levels (Table 1). For Power Scenarios I, II and III, the three improved tests had similar or greater power than AW. For Power Scenarios II and III (i.e. different variances), KS had poor estimated power despite the presence or absence of an outlier. Similar findings about KS are also observed in other parametric distributions (Tables 2 and 4).

**Table 1:** The empirical Type I error rates (× 100) and power (× 100) for the six tests when methylation values were generated from normal distributions without (Outlier=No) or with an outlier (Outlier =Yes). The numbers of non-diseased and diseased samples are (100, 100)

Scenarios	Outlier	*α*(%)	jointLRT	KS	AW	iAW.Lev	iAW.BF	iAW.TM
eqM&eqV	No	5	5.1	3.4	5.1	5.1	5.0	5.1
(Type I error)	No	1	1.0	0.5	1.1	1.0	1.0	1.1
	No	0.5	0.5	0.4	0.6	0.6	0.5	0.6
diffM&eqV	No	5	97.3	95.5	97.1	97.1	97.2	97.2
	No	1	90.2	84.9	89.4	89.8	90.0	89.7
	No	0.5	85.3	75.0	84.3	83.1	83.8	83.6
eqM&diffV	No	5	90.0	25.1	87.3	84.1	83.8	83.8
	No	1	74.3	6.1	65.7	63.5	62.9	62.6
	No	0.5	66.3	2.4	55.2	51.6	52.0	52.5
diffM&diffV	No	5	83.2	63.9	81.0	79.3	79.2	79.3
	No	1	63.7	36.8	59.9	56.9	56.8	56.3
	No	0.5	53.9	24.5	48.8	45.5	46.3	46.2
eqM&eqV	Yes	5	12.2	3.2	3.7	4.8	4.8	4.8
(Type I error)	Yes	1	3.7	0.5	0.5	0.9	0.9	1.0
	Yes	0.5	2.3	0.4	0.3	0.4	0.4	0.4
diffM&eqV	Yes	5	95.6	94.9	98.4	98.1	98.1	98.1
	Yes	1	83.0	86.6	94.5	92.3	92.7	92.4
	Yes	0.5	77.5	76.9	91.0	89.4	90.0	89.3
eqM&diffV	Yes	5	46.3	16.7	54.3	69.3	68.8	68.9
	Yes	1	20.3	5.3	31.5	43.0	43.5	43.2
	Yes	0.5	15.1	2.2	22.8	36.0	36.8	36.1
diffM&diffV	Yes	5	54.6	58.4	75.5	78.4	78.5	78.7
	Yes	1	26.5	38.2	56.9	56.1	57.2	57.0
	Yes	0.5	20.3	25.8	47.5	48.4	50.4	49.1

**Table 2:** The empirical Type I error rates (× 100) and power (× 100) of the six tests when methylation values were generated from Beta distributions. The numbers of non-diseased and diseased samples are (100, 100)

Scenarios	Outlier	*α*(%)	jointLRT	KS	AW	iAW.Lev	iAW.BF	iAW.TM
eqM&eqV	No	5	5.7	3.5	5.4	5.4	5.4	5.5
(Type I error)	No	1	1.5	0.5	1.0	1.1	1.2	1.1
	No	0.5	0.8	0.3	0.5	0.5	0.5	0.5
diffM&eqV	No	5	96.8	94.7	97.5	97.2	97.4	97.4
	No	1	88.4	86.7	91.7	90.5	91.0	90.9
	No	0.5	83.1	77.5	87.8	86.6	87.9	87.4
eqM&diffV	No	5	88.1	18.9	86.8	83.1	82.7	83.0
	No	1	68.6	6.2	65.8	62.2	60.6	61.3
	No	0.5	60.4	2.5	55.6	53.5	52.6	53.2
diffM&diffV	No	5	83.5	64.5	88.6	84.9	85.8	85.9
	No	1	58.1	42.8	70.4	63.0	64.5	64.8
	No	0.5	48.9	30.2	60.6	54.6	57.6	56.8
eqM&eqV	Yes	5	11.0	3.6	3.8	5	4.9	4.9
(Type I error)	Yes	1	3.3	0.6	0.7	1.0	1.0	1.0
	Yes	0.5	1.8	0.3	0.3	0.5	0.5	0.5
diffM&eqV	Yes	5	97.6	95.9	98.8	98.6	98.8	98.7
	Yes	1	89.2	87.7	94.8	93.4	94.0	93.7
	Yes	0.5	82.6	79.6	91.6	89.3	89.9	89.8
eqM&diffV	Yes	5	31.9	15.7	24.9	61.2	59.8	60.6
	Yes	1	11.5	5.1	6.7	33.0	31.3	32.1
	Yes	0.5	6.6	2.0	4.0	23.2	21.3	22.0
diffM&diffV	Yes	5	26.4	59.9	36.6	52.6	53.4	53.6
	Yes	1	8.4	38.3	15.4	24.9	25.7	25.5
	Yes	0.5	4.5	26.0	10.6	16.5	17.2	17.2

Similar findings were also observed for the Beta distribution setting (Table 2). When the Beta distributions of two groups were different in variances (Power Scenarios II and III) and contained outliers, the three improved tests had significantly greater power than AW.

When methylation values were generated from a two-component normal mixture distribution without (Table 3), both iAW.BF and AW had appropriate empirical Type I error rates. However, iAW.Lev and iAW.TM had significantly inflated empirical Type I error rates. Additionally, jointLRT and KS had conservative empirical Type I error rates. Under all Power Scenarios, iAW.BF had greater power than AW and jointLRT. When methylation values were from two-component normal mixture distributions with an outlier, iAW.BF had appropriate simulated Type I error rates at each level. Although iAW.Lev and iAW.TM had increased empirical Type I error rates, they are much smaller than those rates of jointLRT. Whereas KS and AW had conservative empirical Type I error rates. All of the three improved tests had significantly greater power than AW under Power scenarios II (i.e. different variances only) and III (i.e. different means and different variances).

**Table 3:** The empirical Type I error rates (× 100) and power (× 100) for the six tests when methylation values generated from mixtures of two normal distributions. The numbers of non-diseased and diseased samples are (100, 100)

Scenarios	Outlier	*α*(%)	jointLRT	KS	AW	iAW.Lev	iAW.BF	iAW.TM
eqM&eqV	No	5	2.4	3.8	4.9	9.4	5.4	12.3
(Type I error)	No	1	0.4	0.7	0.8	3.2	1.3	4.5
	No	0.5	0.2	0.4	0.4	2.0	0.8	2.8
diffM&eqV	No	5	16.6	58.4	74.9	56.2	87.0	53.6
	No	1	4.0	30.8	55.1	26.6	65.8	25.5
	No	0.5	2.3	25.5	45.1	17.8	53.9	19.6
eqM&diffV	No	5	34.5	98.1	55.1	88.8	57.8	69.9
	No	1	10.5	81.1	36.1	71.6	32.4	47.7
	No	0.5	6.4	72.7	28.9	62.5	23.6	40.5
diffM&diffV	No	5	37.7	98.7	61.1	92.0	68.3	76.4
	No	1	12.0	85.2	41.5	77.2	42.6	54.3
	No	0.5	7.8	78.1	34.0	68.3	32.2	46.7
eqM&eqV	Yes	5	25.0	3.9	2.8	6.5	4.8	8.1
(Type I error)	Yes	1	6.8	0.7	0.4	1.4	1.0	2.1
	Yes	0.5	3.7	0.4	0.2	0.7	0.6	1.3
diffM&eqV	Yes	5	4.2	59.4	16.3	21.5	78.1	34.9
	Yes	1	1.1	32.1	5.1	5.2	55.7	9.8
	Yes	0.5	0.5	26.5	3.3	3.5	44.7	5.2
eqM&diffV	Yes	5	0.6	97.4	14.4	80.2	49.6	63.3
	Yes	1	0.1	79.5	5.1	59.8	27.4	39.4
	Yes	0.5	0.0	71.2	3.5	54.1	19.7	31.5
diffM&diffV	Yes	5	1.0	98.1	19.5	84.6	61.0	71.1
	Yes	1	0.2	83.6	7.5	65.7	37.5	47.0
	Yes	0.5	0.1	76.8	5.6	60.1	27.9	38.1

**Table 4:** The empirical Type I error rates (× 100) and power (× 100) for the six tests when methylation values generated from chi-squared distributions. The numbers of non-diseased and diseased samples are (100, 100)

Scenarios	Outlier	*α*(%)	jointLRT	KS	AW	iAW.Lev	iAW.BF	iAW.TM
eqM&eqV	No	5	13.8	4.2	5.0	6.3	5.3	5.2
(Type I errror)	No	1	6.3	0.7	0.9	1.5	1.3	1.2
	No	0.5	4.4	0.4	0.4	0.8	0.5	0.5
diffM&eqV	No	5	90.2	99.7	99.8	99.6	99.9	99.9
	No	1	53.8	97.1	99.0	97.1	99.4	99.4
	No	0.5	40.9	95.9	98.1	94.9	99.2	99.0
eqM&diffV	No	5	18.6	10.2	29.2	29.6	35.4	34.6
	No	1	5.8	2.1	10.3	11.4	14.7	15.0
	No	0.5	3.9	1.3	6.9	7.0	11.1	10.4
diffM&diffV	No	5	18.4	42.2	59.9	54.9	70.6	69.0
	No	1	3.7	17.9	35.7	27.5	45.5	43.8
	No	0.5	2.1	13.9	27.9	18.9	38.9	35.6
eqM&eqV	Yes	5	20.1	4.0	4.8	6.7	5.5	5.3
(Type I error)	Yes	1	10.3	0.7	0.7	1.7	1.1	1.1
	Yes	0.5	7.8	0.5	0.2	0.8	0.5	0.5
diffM&eqV	Yes	5	67.9	99.5	99.9	99.4	99.9	99.9
	Yes	1	23.7	96.5	99.1	96.4	99.4	99.3
	Yes	0.5	12.9	95.0	98.7	94.0	99.0	98.8
eqM&diffV	Yes	5	27.5	9.5	34.0	39.7	41.0	41.5
	Yes	1	9.9	1.8	11.9	16.6	19.3	19.0
	Yes	0.5	6.1	1.1	7.3	11.2	14.0	13.7
diffM&diffV	Yes	5	21.9	39.8	65.2	60.4	73.2	72.1
	Yes	1	6.3	16.3	39.9	31.7	49.7	47.7
	Yes	0.5	3.4	12.2	32.5	23.9	41.8	39.7

When methylation values were generated from a chi-squared distribution without (Table 4), iAW.BF, iAW.TM and AW kept empirical Type I error rates well, though iAW.Lev presented increased empirical Type I error rates. While jointLRT had inflated empirical Type I error rates, and KS has rather conservative empirical Type I error rates. For Power scenarios II and III (i.e. different variances), iAW.BF and iAW.TM had significantly greater power than AW. Besides, iAW.Lev had similar power to AW for three power scenarios. When methylation values were generated from chi-squared distribution with an outlier, the performances of all tests are similar except that AW had conservative empirical Type I error rates.

From the results of the four tables, we found that iAW.BF could control empirical Type I error rates well and have similar or greater power than AW under all scenarios including the existence of outliers, skewed distributions and mixtures of two normal distributions. Except for the scenarios of mixtures of two normal distributions, iAW.Lev and iAW.TM can maintain empirical Type I error rates at proper levels and had similar or greater power than AW. In comparison, AW can keep appropriate empirical Type I error rates for any parametric distributions as designed without outliers. But when the methylation values were generated from a distribution with an outlier, AW tended to have conservative empirical Type I error rates and smaller estimated power. The jointLRT, on the other hand, only performed best for methylation values generated from normal distributions without outliers. KS can keep conservative empirical Type I error rates under all scenarios, and it had poor estimated power in many scenarios.

Simulation studies were also conducted when sample size was moderate (50, 50) or small (20, 20). The results are provided in Additional file 1: Tables S2-S9). We observed that empirical Type I error rates increased and power decreased when sample size decreased from 100 to 50 subjects per group. Furthermore, the three improved joint score tests still performed significantly better than AW under moderate or small sample size.

### Real data analyses

We applied all six statistical tests to three publicly available DNA methylation data sets (GSE37020 [16], GSE20080 [17] and GSE107080 [18]) from Gene Expression Omnibus (GEO)(*www.ncbi.nlm.nih.gov/geo*).

GSE37020 and GSE20080 used Illumina HumanMethylation27 (HM27k) platform to produce DNA methylation profiles for 27,578 CpG sites. Both data sets measured cervical smear samples collected from normal histology (regarded as normal samples) and changed tissues with cervical intraepithelial neoplasia of grade 2 or higher (CIN2+) (CIN2+ samples). GSE37020 contains 24 normal samples and 24 CIN2+ samples, while GSE20080 contains 30 normal samples and 18 CIN2+ samples. GSE107080 contained DNA methylation profiles of about 850K sites measured from whole blood samples using Illumina Infinium MethylationEPIC (EPIC) platform. GSE107080 included 100 individuals with illicit drug injection and hepatitis C type virus (IDU+/HCV+) and 305 individuals without illicit drug injection and hepatitis C type virus (IDU-/HCV-). All the individuals are recruited from a well-established longitudinal cohort, Veteran Aging Cohort Study.

For GSE37020 and GSE20080, we excluded CpG sites residing near SNPs or with missing values. Quantile plots and principal component analysis did not show obvious and suspicious patterns (for details please refer to [4]). We then obtained residuals of samples after regressing out the effect of age from DNA methylation levels. We re-did the principal component analysis on the adjusted data and did not find any obvious patterns (see Additional file 1: Figure S2). After data quality control and preprocessing (for details please refer to [4]), there were 22,859 CpG sites appearing in both cleaned data sets.

We used cleaned GSE37020 as the discovery set and cleaned GSE20080 as the validation set to detect CpG sites differentially methylated (DM) or differentially variable (DV) between CIN2+ samples and normal samples. For a given CpG site in a given data set, we applied each of the six joint tests to test for equalities of both means and variances. For a given joint test, we claimed a CpG site in the analysis of GSE37020 as significant methylation candidate (different in means or variances) if the false discovery rate (FDR) [19] adjusted *p*-value for the CpG site is less than 0.05. The function *p.adjust* in the statistical software *R* was used to calculate FDR-adjusted *p*-value. For a significant site in the analysis of GSE37020, if the corresponding un-adjusted *p*-value in the analysis of GSE20080 is less than 0.05 and the difference directions of means and variances are consistent between the two data sets, then we claim that the significance in the analysis of GSE37020 is truly validated in the analysis of GSE20080. We use the differences of medians and mean absolute deviations between cases and controls to evaluate the directions.

For HM27k data set GSE37020, the numbers of significant CpG sites (i.e., CpG sites with FDR-adjusted *p*-value < 0.05) obtained by the 6 joint tests are 4556 (jointLRT), 1288 (KS), 1850 (AW), 2041 (iAW.Lev), 1843 (iAW.BF) and 1838 (iAW.TM). And the truly validated CpG sites are 1705 (jointLRT), 47 (KS), 220 (AW), 666 (iAW.Lev), 296 (iAW.BF) and 342 (iAW.TM).

Table 5 presents the numbers/proportions of truly and falsely validated significant CpG sites. The three improved joint score tests have higher true validation ratios than joint LRT, KS test, and AW test. Among all the tests, iAW.Lev had the highest true validation rate (89.2%) and lowest false validation rate (10.8%), followed by iAW.TM and iAW.BF. And we also applied the 6 joint tests on the adjusted data sets, the performances of them are similar (see Additional file 1: Table S1).

**Table 5:** The performances of 6 joint tests based on HM27k data GSE37020 and GSE20080

Test	nSig	nValidation	nTV	pTV(%)	nFV	pFV(%)
JointLRT	4556	2213	1705	77.0	508	23.0
KS	1288	60	47	78.3	13	21.7
AW	1850	262	220	84.0	42	16.0
iAW.Lev	2041	747	666	89.2	81	10.8
iAW.BF	1843	339	296	87.3	43	12.7
iAW.TM	1838	387	342	88.4	45	11.6

^nSig^: the number of significant CpG sites detected in GSE37020 based on FDR adjusted *p*-value < 0.05;

^nValidation^: the number of validated CpG sites in GSE20080 based on unadjusted *p*-value < 0.05;

^nTV^: the number of truly validated CpG sites with the same difference directions in means and variances between the two groups;

^pTV^: $=\frac {nTV}{nValidation}$, the proportion of significant CpG sites detected in GSE37020 and truly validated in GSE20080;

^nFV^: the number of falsely validated CpG sites in GSE20080 with inconsistent difference direction in means or variances between the two groups;

^pFV^: $=\frac {nFV}{nValidation}$, the proportion of significant CpG sites detected in GSE37020 but falsely validated in GSE20080

**Fig. 1:**
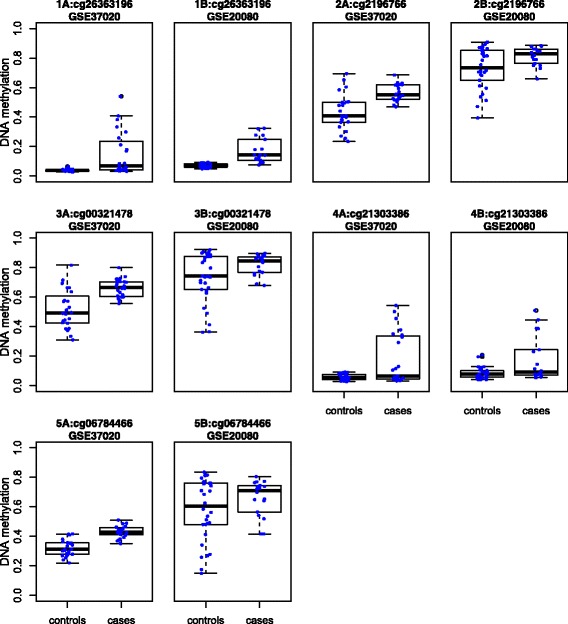
Paired parallel boxplots of DNA methylation levels (y axis) versus case-control status (x axis) for the 5 unique top CpG sites acquired by the 6 joint tests based on HM27k data sets. The dots indicate subjects.1A and 1B are for cg26363196 (jointLRT). 2A and 2B are for cg2196766 (KS). 3A and 3B are for cg00321478 (AW). 4A and 4B are for cg21303386 (iAW.Lev). 5A and 5B are for cg06784466 (iAW.BF, iAW.TM). 1A,2A,3A,4A,5A are based on GSE37020. 1B,2B,3B,4B,5B are based on GSE20080

Figure 1 showed the parallel boxplots of DNA methylation levels versus case-control status for the top CpG site (i.e. having the smallest *p*-value among those truly validated CpG sites for testing homogeneity of means and variances simultaneously) obtained by each of the 6 joint tests. All these top CpG sites were validated in GSE20080. It has been found that the high incidence of cervical lesions is associated to the genes ST6GALNAC3, CRB1 and RGS7, where cg26363196 (jointLRT), cg00321478 (AW) and cg21303386 (iAW.Lev) might reside [20, 21]. Furthermore, the gene PRRG2, where cg2196766 (KS) might reside, is involved in signal transduction pathway, which might be a novel biomarker for CIN2+ diagnosis [22]. And the gene FPRL2, where cg06784466 (iAW.BF, iAW.TM) might reside, are related to innate immunity and host defense mechanisms [23].

For GSE107080, we downloaded the processed data set from GEO database [18]. We first removed the CpG sites with at least one missing value or with probe name using “ch” as the prefix. Secondly, CpG sites with detection *p*-values larger than or equal to 10^−12^ are discarded. There are 378,808 CpG sites in the cleaned data set. We drew the plot of quantiles across arrays and did a principal component analysis for the cleaned GSE107080 data set. The results did not show any obvious patterns (see Additional file 1: Figure S3). Additionally, we regressed out the effects of age and cell type compositions and obtained the residuals. There are 378,808 CpG sites and 309 samples (cases: 95 and controls: 295) left in the data set after the adjustment. Results from the principal component analysis on the adjusted data did not show any obvious patterns (see Additional file 1: Figure S4).

For the EPIC data set GSE107080, the samples were randomly split into two sets with approximately equal size (due to odd numbers of cases and controls) as the training set and the validation set. The training set contained 148 controls (IDU-/HCV-) and 48 cases (IDU+/HCV+), and the validation set contained 147 controls and 47 cases. We use the similar method as above to determine if the significance of a CpG site is truly validated.

For GSE107080, the numbers of significant CpG sites (i.e., CpG sites with FDR-adjusted *p*-value < 0.05) obtained by the 6 joint tests in the training set are 51,994 (jointLRT), 10 (KS), 12 (AW), 709 (iAW.Lev), 22 (iAW.BF) and 22 (iAW.TM). And the corresponding numbers of validated CpG sites in the validation set (i.e., CpG sites with unadjusted *p*-value < 0.05) are 19,806 (jointLRT), 3 (KS), 5 (AW), 201 (iAW.Lev), 7 (iAW.BF) and 9 (iAW.TM). After checking the difference directions, the truly validated CpG sites are 5652 (jointLRT), 1 (KS), 2 (AW), 89 (iAW.Lev), 4 (iAW.BF) and 5 (iAW.TM).

Table 6 presents the numbers/proportions of truly and falsely validated significant CpG sites based on GSE107080. The three improved tests have higher true validation ratios than joint LRT, KS and AW tests. Among the three improved tests, iAW.BF and iAW.TM have more than ten percent higher proportion of true validation than AW.

**Table 6:** The performances of 6 joint tests based on EPIC data GSE107080

Test	nSig	nValidation	nTV	pTV(%)	nFV	pFV(%)
JointLRT	51994	19806	5652	28.5	14154	71.5
KS	10	3	1	33.3	2	66.7
AW	12	5	2	40.0	3	60.0
iAW.Lev	709	201	89	44.3	112	55.7
iAW.BF	22	7	4	57.1	3	42.9
iAW.TM	22	9	5	55.6	4	44.4

^nSig^: the number of significant CpG sites detected in the training set of GSE107080 based on FDR adjusted *p*-value < 0.05;

^nValidation^: the number of validated CpG sites in the validation set of GSE107080 based on unadjusted *p*-value < 0.05;

^nTV^: the number of truly validated CpG sites with the same difference directions in means and variances between the two groups;

^pTV^: $=\frac {nTV}{nValidation}$, the proportion of significant CpG sites detected in the training set and truly validated in the validation set;

^nFV^: the number of falsely validated CpG sites in validation set with inconsistent difference direction in means or variances between the two groups;

^pFV^: $=\frac {nFV}{nValidation}$, the proportion of significant CpG sites detected in the training set but falsely validated in the validation set

## Discussion

The three improved joint score tests are derived from generalized linear model framework as AW. Thus they maintain the strengths of AW in terms of efficiency. Furthermore, the three improved tests use absolute deviation instead of squared deviation used by AW to enhance the robustness. For skewed methylation distributions or distributions with outliers, squared deviation used by AW can be enormously affected by extreme values and leads to erroneous results. Thus AW tends to have conservative empirical Type I error rates and smaller power in some scenarios. Our proposed methods rectify this problem and can maintain good power even if the distribution is skewed or contains one or more outliers. Besides, when compared to squared deviation, absolute deviation retains the same magnitude of the original measurement scales and consequently more interpretable. The iAW.Lev tends to have inflated empirical Type I error rates under skewed and mixture distributions. In comparison, iAW.BF and iAW.TM employ median and trimmed mean as central tendency respectively to calculate absolute deviation. Both of them are robust and can minimize the impact of outliers and skewed distributions in evaluating the overall dispersion of the sample data.

The performance of the jointLRT was highly dependent on the validity of normality assumptions. However, the empirical distribution of methylation data often demonstrates skewness and presence of outlying observations. The KS test was inclined to have conservative empirical Type I error rates and lowest power under many scenarios. Therefore it might not be suitable for DNA methylation analysis as expected.

We would like to address one limitation of our simulation studies. Since the analytical form of the underlying probability distribution of methylation data is rarely known, we have applied various settings in an attempt to mimic the reality. We also tried to evaluate our methods in four different aspects. However, our simulation study might not cover all cases that one might encounter in reality. Nevertheless, the results from real data analyses provide strong evidence to support the thesis that our proposed tests are in general more robust in comparison with the AW test.

Another remark is that the AW test and our improved tests are motivated and connected to the logistic regression. Potentially, these tests could be applied for prediction of disease. The difference of performances of our three proposed tests could be disease-related. In other words, one test might be more suitable for one specific type of disease.

We would also like to make some remarks about the important issue of striking a delicate balance between controlling the false positive rate and increasing testing power. In genomic data analysis, controlling false positive is an important issue. This is why the adjustment of *p*-values is required to control for multiple testing that could result in highly inflated type I error rates. However, when sample size is small (e.g., in pilot studies), we usually have to make some assumptions in order to carry out statistical inference. In this case, we can make the normality assumption and apply an F-test to detect differentially variable CpG sites.

Finally, we would like to remark that we can further validate the differentially methylated/variable (DM/DV) CpG sites, which were identified in our real data analysis, by technical validation. In the technical validation, we can use pyrosequencing technology to measure more accurately the DNA methylation levels of the identified CpG sites for a subset of cases and controls. If one specific CpG site is detected as DM/DV based on the pyrosequenced data, then we gain more evidence that this CpG site is DM/DV. Pathway enrichment analysis could also provide further evidence that the identified CpG sites are relevant to the disease of interest.

## Conclusion

Results from simulation studies and real data analyses have demonstrated that the three proposed joint score tests performed better than the existing methods (AW, jointLRT, and KS) for testing equal means and variances simultaneously when methylation levels contained outliers or had different variances between diseased and non-diseased samples.

In general, iAW.BF was the most robust method in terms of power among all the scenarios considered in our simulation study. It also has significantly better performance when compared with the AW test. For the cases of mixtures of two normal distributions, iAW.Lev and iAW.TM performed similarly to or better than AW. In addition, the proposed tests can be easily applied to very large methylation data sets, eg. data sets from the platforms HM27k and EPIC.

## Additional file

Additional file 1 Supplementary Materials to: Robust Joint Score Tests in the Application of DNA Methylation Data Analysis. This file contains: A. Derivation of the asymptotic distribution of the AW test statistic; B. Quality control and data preprocessing for three real data sets; C. Additional simulation results. (PDF 365 kb)

## Abbreviations

AW: Ahn and Wang’s joint score test
CpG: a type of DNA methylation mark
CIN2+: cervical intraepithelial neoplasia of grade 2 or higher
DM: differently methylated
DV: differently variable
diffM: Different means
diffV: Different variances
EPIC: Illumina Infinium MethylationEPIC
eqM: Equal means
eqV: Equal variances
GEO: Gene Expression Omnibus
HCV: hepatitis C type virus
HM27k: Illumina HumanMethylation27
HM450k: Illumina HumanMethylation450
iAW.Lev: improved AW joint score test based on absolute deviation from mean
iAW.BF: improved AW joint score test based on absolute deviation from median
iAW.TM: improved AW joint score test based on absolute deviation from trimmed mean
IDU: illicit drug injection
jointLRT: Joint likelihood ratio test
KS: Kolmogorov-Smirnov test
SNP: single nucleotide polymorphism
