# Supplementary material for: Robust joint score tests in the application of DNA methylation data analysis
Source: BMC Bioinformatics. 2018 May 18;19:174. doi: 10.1186/s12859-018-2185-3 (PMC5960098; doi:10.1186/s12859-018-2185-3)
Supplement: Supplementary file 1 — Supplementary Materials to: Robust Joint Score Tests in the Application of DNA Methylation Data Analysis. This file contains: A. Derivation of the asymptotic distribution of the AW test statistic; B. Quality control and data preprocessing for three real data sets; C. Additional simulation results. (PDF 365 kb) [file 12859_2018_2185_MOESM1_ESM.pdf]

Supplementary Materials to: Robust Joint Score Tests in the Application of DNA Methylation Data Analysis

Xuan Li[1], Yuejiao Fu[2,*], Xiaogang Wang[3], Weiliang Qiu[4]

[1,2,3] Department of Mathematics and Statistics, York University, 4700 Keele Street, Toronto, ON, M3J1P3, Canada

[1] Email: lixuan@mathstat.yorku.ca

[2] Email: yuejiao@mathstat.yorku.ca

[3] Email: stevenw@mathstat.yorku.ca

[4] Channing Division of Network Medicine, Brigham and Women's Hospital, Harvard Medical School, 181 Longwood Avenue, Boston, 02115, USA

[4] Email: stwxq@channing.harvard.edu

[*] Corresponding author

A Derivation of the asymptotic distribution of the AW test statistic

Ahn and Wang (2013) [1] proposed a joint score test (denoted as AW test) to detect "methylation markers of disease". This joint test evaluates equalities of means and variances simultaneously. But Ahn and Wang did not provide the derivation of the asymptotic distribution for this joint score test. Now we fill this gap and propose three improved joint score tests.

Let X_i and Y_i denote the methylation value and the disease status of subject i, where $i = 1, 2, ..., n$, with $n = n_0 + n_1$, n_0 is the number of the non-diseased subjects (controls, $Y_i = 0$) and n_1 is the number of the diseased subjects (cases, $Y_i = 1$). To detect methylation loci

that are relevant to the disease based on means and variances, the corresponding hypothesis is considered as $H_0 : \mu_0 = \mu_1$ and $\sigma_0^2 = \sigma_1^2$ versus $H_1 : \mu_0 \neq \mu_1$ or $\sigma_0^2 \neq \sigma_1^2$, in which μ_0 and μ_1 are means of methylation levels for controls and cases, respectively, and σ_0^2 and σ_1^2 are the corresponding variances.

Instead of directly testing above hypothesis, Ahn and Wang (2013) proposed to test $H_0' : \beta_1 = \beta_2 = 0$ versus $H_a' : \beta_1 \neq 0$ or $\beta_2 \neq 0$, where β_1 and β_2 are the regression coefficients of the following logistic regression:

$$logit[Pr(Y_i = 1|x_i, z_i)] = \beta_0 + \beta_1 x_i + \beta_2 z_i, \tag{A1}$$

and z_i is the within-group squared deviation for subject i, which is defined as

$$z_i = \begin{cases} (x_i - \bar{x}_1)^2, \text{ if } Y_i = 1 \\ (x_i - \bar{x}_0)^2, \text{ if } Y_i = 0, \end{cases} \tag{A2}$$

and $\bar{x}_1 = \sum_{i=1}^n x_i I[y_i = 1]/n_1$ and $\bar{x}_0 = \sum_{i=1}^n x_i I[y_i = 0]/n_0$ are the sample means for cases and controls.

The log-likelihood function of the logistic regression (A1) is

$$l(\mathbf{\Theta}) = \sum_{i=1}^n y_i(\beta_0 + \beta_1 x_i + \beta_2 z_i) - \log[1 + exp(\beta_0 + \beta_1 x_i + \beta_2 z_i)],$$

where $\mathbf{\Theta} = (\beta_0, \beta_1, \beta_2)^T$. The score statistics are partial derivatives of the log-likelihood function with respect ot the parameters of interest, evaluated at the values postulated by the null hypothesis $H_0' : \beta_1 = \beta_2 = 0$.

We have

$$\frac{\partial l(\boldsymbol{\Theta})}{\partial \beta_0} = \sum_{i=1}^{n} (y_i - \pi_i),$$
$$\frac{\partial l(\boldsymbol{\Theta})}{\partial \beta_1} = \sum_{i=1}^{n} x_i (y_i - \pi_i),$$
$$\frac{\partial l(\boldsymbol{\Theta})}{\partial \beta_2} = \sum_{i=1}^{n} z_i (y_i - \pi_i),$$

where

$$\pi_i = Pr(Y_i = 1 | x_i, z_i) = \frac{\exp(\beta_0 + \beta_1 x_i + \beta_2 z_i)}{1 + \exp(\beta_0 + \beta_1 x_i + \beta_2 z_i)}.$$

Under $H_0' : \beta_1 = \beta_2 = 0$,

$$\pi_i \overset{H_0'}{=} \frac{\exp(\beta_0)}{1 + \exp(\beta_0)} \equiv \pi_0.$$

Let $\partial l(\boldsymbol{\Theta}) / \partial \beta_0 = 0$ under H_0'. We got an estimate of π_0:

$$\hat{\pi}_0 = \bar{y} = \sum_{i=1}^{n} y_i / n.$$

Hence, the score statistics are

$$U_1 = \left. \frac{\partial l(\boldsymbol{\Theta})}{\partial \beta_1} \right|_{\pi_0 = \bar{y}, \beta_1 = \beta_2 = 0} = \sum_{i=1}^{n} x_i (y_i - \bar{y}),$$
$$U_2 = \left. \frac{\partial l(\boldsymbol{\Theta})}{\partial \beta_2} \right|_{\pi_0 = \bar{y}, \beta_1 = \beta_2 = 0} = \sum_{i=1}^{n} z_i (y_i - \bar{y}).$$

By simple alegra and the fact that $y_i = 1$ or 0, we can get

$$
\begin{aligned}
U_1 =& \sum_{i=1}^{n} x_i(y_i - \bar{y}) \\
=& \sum_{i=1}^{n} x_i y_i - \bar{y} \sum_{i=1}^{n} x_i \\
=& n_1 \bar{x}_1 - \frac{n_1}{n} \left(n_1 \bar{x}_1 + n_0 \bar{x}_0 \right) \\
=& \frac{n_1 n_0}{n} \left(\bar{x}_1 - \bar{x}_0 \right), \\
=& \frac{n_1 n_0}{n} \left(\hat{\mu}_1 - \hat{\mu}_0 \right).
\end{aligned}
$$

Similarly, we have

$$
\begin{aligned}
U_2 =& \sum_{i=1}^{n} z_i(y_i - \bar{y}) \\
=& \frac{n_1 n_0}{n} \left(\bar{z}_1 - \bar{z}_0 \right) \\
=& \frac{n_1 n_0}{n} \left(\frac{1}{n_1} \sum_{i=1}^{n} (x_i - \bar{x}_1)^2 I[y_i = 1] + \frac{1}{n_0} \sum_{i=1}^{n} (x_i - \bar{x}_0)^2 I[y_i = 0] \right) \\
=& \frac{n_1 n_0}{n} \left(\hat{\sigma}_1^2 - \hat{\sigma}_0^2 \right).
\end{aligned}
$$

Hence, large value of U_1^2 indicates large difference between $\hat{\mu}_1$ and $\hat{\mu}_0$. And large value of U_2^2 indicates large difference between $\hat{\sigma}_1^2$ and $\hat{\sigma}_0^2$.

Ahn and Wang's (2013) joint test statistic $T = \mathbf{U}^T \hat{\mathbf{\Sigma}}^{-1} \mathbf{U}$ is the quadratic form of the two score statistics U_1 and U_2 for the above logistic regression, where $\mathbf{U} = (U_1, U_2)^T$ and $\hat{\mathbf{\Sigma}}$ is the estimate of the covariance matrix $Cov(\mathbf{U})$.

Note that in logistic regression (A1), y_i are random variables, given x_i and z_i are known

(i.e., non-random). We can get

$$E(U_1) = \sum_{i=1}^{n} x_i E(y_i - \bar{y}) = 0,$$
$$E(U_2) = \sum_{i=1}^{n} z_i E(y_i - \bar{y}) = 0.$$

The above equalities are true, no matter whether the null hypothesis H_0' holds or not. Hence, we have

$$\begin{aligned} Cov(\mathbf{U}) =& E\left(\mathbf{U}\mathbf{U}^T\right) - [E(\mathbf{U})][E(\mathbf{U})]^T \\ =& E\left(\mathbf{U}\mathbf{U}^T\right) \\ =& \begin{pmatrix} E(U_1^2) & E(U_1U_2) \\ E(U_1U_2) & E(U_2^2) \end{pmatrix}. \end{aligned} \tag{A3}$$

We can get

$$\begin{aligned} U_1^2 =& \left[\sum_{i=1}^{n} x_i (y_i - \bar{y})\right]^2 \\ =& \left[\sum_{i=1}^{n} x_i y_i - \bar{y}\sum_{i=1}^{n} x_i\right]^2 \\ =& \left(\sum_{i=1}^{n} x_i y_i\right)^2 + \bar{y}^2\left(\sum_{i=1}^{n} x_i\right)^2 - 2\left(\sum_{i=1}^{n} x_i y_i\right)\left(\bar{y}\sum_{j=1}^{n} x_j\right) \\ =& \sum_{i=1}^{n} x_i y_i \sum_{j=1}^{n} x_j y_j + \bar{y}^2\left(\sum_{i=1}^{n} x_i\right)^2 - 2\left(\sum_{j=1}^{n} x_j\right)\left(\sum_{i=1}^{n} x_i y_i \bar{y}\right) \\ =& \sum_{i=1}^{n}\sum_{j=1}^{n} x_i x_j y_i y_j + \bar{y}^2\left(\sum_{i=1}^{n} x_i\right)^2 - 2\left(\sum_{j=1}^{n} x_j\right)\left(\sum_{i=1}^{n} x_i y_i \bar{y}\right) \end{aligned}$$

Note that $y_i^2 = y_i$. We have

$$
\begin{aligned}
& \mathrm{E}\left(\sum_{i=1}^{n}\sum_{j=1}^{n} x_i x_j y_i y_j\right) \\
=& \sum_{i=1}^{n}\sum_{j=1}^{n} x_i x_j \mathrm{E}\left(y_i y_j\right) \\
=& \sum_{i=1}^{n} x_i^2 \mathrm{E}\left(y_i^2\right) + \sum_{i \neq j} x_i x_j \mathrm{E}\left(y_i\right) \mathrm{E}\left(y_j\right) \\
=& \sum_{i=1}^{n} x_i^2 \mathrm{E}\left(y_i\right) + \sum_{i \neq j} x_i x_j \pi_i \pi_j \\
=& \sum_{i=1}^{n} x_i^2 \pi_i + \sum_{i \neq j} x_i x_j \pi_i \pi_j \\
=& \sum_{i=1}^{n} x_i^2 \pi_i - \sum_{i=1}^{n} x_i^2 \pi_i^2 + \sum_{i=1}^{n} x_i^2 \pi_i^2 + \sum_{i \neq j} x_i x_j \pi_i \pi_j \\
=& \sum_{i=1}^{n} x_i^2 \pi_i \left(1 - \pi_i\right) \sum_{i=1}^{n}\sum_{j=1}^{n} x_i x_j \pi_i \pi_j \\
=& \sum_{i=1}^{n} x_i^2 \pi_i \left(1 - \pi_i\right) + \left(\sum_{i=1}^{n} x_i \pi_i\right)^2 .
\end{aligned}
$$

We also have

$$
\begin{aligned}
\mathrm{E}\left(\bar{y}\right)^2 =& \mathrm{Var}\left(\bar{y}\right) + \left[\mathrm{E}\left(\bar{y}\right)\right]^2 \\
=& \frac{1}{n^2}\sum_{i=1}^{n} \mathrm{Var}\left(y_i\right) + \left[\frac{1}{n}\sum_{i=1}^{n} \pi_i\right]^2 \\
=& \frac{1}{n^2}\sum_{i=1}^{n} \pi_i \left(1 - \pi_i\right) + \left[\frac{1}{n}\sum_{i=1}^{n} \pi_i\right]^2
\end{aligned}
\tag{A4}
$$

And

$$
\begin{aligned}
\mathrm{E}\left(\sum_{i=1}^{n} x_i y_i \bar{y}\right) &= \sum_{i=1}^{n} x_i \mathrm{E}\left(y_i \bar{y}\right) \\
&= \sum_{i=1}^{n} x_i \mathrm{E}\left[y_i \frac{1}{n} \sum_{j=1}^{n} y_j\right] \\
&= \frac{1}{n} \sum_{i=1}^{n} x_i \sum_{j=1}^{n} \mathrm{E}\left(y_i y_j\right) \\
&= \frac{1}{n} \sum_{i=1}^{n} x_i \left[\sum_{j=1}^{n} \mathrm{E}\left(y_i y_j\right)\right] \\
&= \frac{1}{n} \sum_{i=1}^{n} x_i \left[\mathrm{E}\left(y_i^2\right) + \sum_{j=1, j\neq i}^{n} \mathrm{E}\left(y_i\right) \mathrm{E}\left(y_j\right)\right] \\
&= \frac{1}{n} \sum_{i=1}^{n} x_i \left[\mathrm{E}\left(y_i\right) + \sum_{j=1, j\neq i}^{n} \pi_i \pi_j\right] \\
&= \frac{1}{n} \sum_{i=1}^{n} x_i \left[\pi_i + \sum_{j=1, j\neq i}^{n} \pi_i \pi_j\right] \\
&= \frac{1}{n} \sum_{i=1}^{n} x_i \left[\pi_i - \pi_i^2 + \pi_i^2 + \sum_{j=1, j\neq i}^{n} \pi_i \pi_j\right] \\
&= \frac{1}{n} \sum_{i=1}^{n} x_i \left[\pi_i \left(1 - \pi_i\right) + \sum_{j=1}^{n} \pi_i \pi_j\right] \\
&= \frac{1}{n} \sum_{i=1}^{n} x_i \left[\pi_i \left(1 - \pi_i\right) + \pi_i \sum_{j=1}^{n} \pi_j\right]
\end{aligned}
$$

Hence,

$$
\begin{aligned}
\mathrm{E}\left(U_1^2\right) = &\sum_{i=1}^{n} \sum_{j=1}^{n} x_i x_j \mathrm{E}\left(y_i y_j\right) + \mathrm{E}\left(\bar{y}^2\right) \left(\sum_{i=1}^{n} x_i\right)^2 - 2 \left(\sum_{j=1}^{n} x_j\right) \sum_{i=1}^{n} x_i \mathrm{E}\left(y_i \bar{y}\right) \\
= &\sum_{i=1}^{n} x_i^2 \pi_i \left(1 - \pi_i\right) + \left(\sum_{i=1}^{n} x_i \pi_i\right)^2 \\
&+ \left[\frac{1}{n^2} \sum_{i=1}^{n} \pi_i \left(1 - \pi_i\right) + \left(\frac{1}{n} \sum_{i=1}^{n} \pi_i\right)^2\right] \left(\sum_{i=1}^{n} x_i\right)^2 \\
&- 2 \left(\sum_{j=1}^{n} x_j\right) \frac{1}{n} \sum_{i=1}^{n} x_i \left[\pi_i \left(1 - \pi_i\right) + \pi_i \sum_{j=1}^{n} \pi_j\right]
\end{aligned}
\tag{A5}
$$

Under $H_0' : \beta_1 = \beta_2 = 0$, we can estimate π_0 by $\bar{y} = n_1/n$ and can estimate $\mathrm{E}\ (U_1)^2$ by

$$
\begin{aligned}
\widehat{\mathrm{E}}\ (U_1|H_0')^2 =& \sum_{i=1}^{n} x_i^2 \frac{n_1}{n}\left(1-\frac{n_1}{n}\right) + \left(\sum_{i=1}^{n} x_i \frac{n_1}{n}\right)^2 \\
&+ \left[\frac{1}{n^2}\sum_{i=1}^{n}\frac{n_1}{n}\left(1-\frac{n_1}{n}\right) + \left(\frac{1}{n}\sum_{i=1}^{n}\frac{n_1}{n}\right)^2\right]\left(\sum_{i=1}^{n} x_i\right)^2 \\
&- 2\left(\sum_{j=1}^{n} x_j\right)\frac{1}{n}\sum_{i=1}^{n} x_i\left[\frac{n_1}{n}\left(1-\frac{n_1}{n}\right) + \frac{n_1}{n}\sum_{j=1}^{n}\frac{n_1}{n}\right] \\
=& \frac{n_1}{n}\frac{n_0}{n}\sum_{i=1}^{n} x_i^2 + \frac{n_1^2}{n^2}\left(\sum_{i=1}^{n} x_i\right)^2 \\
&+ \left[\frac{1}{n^2}\frac{n_1}{n}\frac{n_0}{n}n + \frac{1}{n^2}\frac{n_1^2}{n^2}n^2\right]\left(\sum_{i=1}^{n} x_i\right)^2 \\
&- \frac{2}{n}\left(\sum_{j=1}^{n} x_j\right)^2\left[\frac{n_1 n_0}{n^2} + \frac{n_1^2}{n^2}n\right] \\
=& \frac{n_1}{n}\frac{n_0}{n}\sum_{i=1}^{n} x_i^2 + \left(\sum_{i=1}^{n} x_i\right)^2\left[\frac{n_1^2}{n^2} + \frac{n_1 n_0}{n^3} + \frac{n_1^2}{n^2} - 2\frac{n_1 n_0}{n^3} - 2\frac{n_1^2}{n^2}\right] \\
=& \frac{n_1}{n}\frac{n_0}{n}\sum_{i=1}^{n} x_i^2 - \frac{n_1 n_0}{n^3}\left(\sum_{i=1}^{n} x_i\right)^2 \\
=& \frac{n_1}{n}\frac{n_0}{n}\left[\sum_{i=1}^{n} x_i^2 - \frac{1}{n}\left(\sum_{i=1}^{n} x_i\right)^2\right] \\
=& \bar{y}\,(1-\bar{y})\sum_{i=1}^{n}(x_i-\bar{x})^2 .
\end{aligned}
$$

That is,

$$
\widehat{Var(U_1|H_0')} = \widehat{E(U_1^2|H_0')} = \bar{y}\,(1-\bar{y})\sum_{i=1}^{n}(x_i-\bar{x})^2 .
$$

Similarly, we can estimate $Var(U_2)$ by

$$
\widehat{Var(U_2|H_0')} = \widehat{E(U_2^2|H_0')} = \bar{y}\,(1-\bar{y})\sum_{i=1}^{n}(z_i-\bar{z})^2 .
$$

Next, we calculate $E(U_1U_2)$.

$$
\begin{aligned}
\mathrm{E}\left[U_1 U_2\right] =& \mathrm{E}\left[\sum_{i=1}^{n} x_i \left(y_i - \bar{y}\right) \sum_{j=1}^{n} z_j \left(y_j - \bar{y}\right)\right] \\
=& \sum_{i=1}^{n} \sum_{j=1}^{n} x_i z_j \mathrm{E}\left[\left(y_i - \bar{y}\right)\left(y_j - \bar{y}\right)\right] \\
=& \sum_{i=j} x_i z_i \mathrm{E}\left[\left(y_i - \bar{y}\right)^2\right] \\
&+ \sum_{i \neq j} x_i z_j \mathrm{E}\left[\left(y_i - \bar{y}\right)\left(y_j - \bar{y}\right)\right]
\end{aligned}
$$

Note that $y_i^2 = y_i$ since y_i is binary variable taking values 1 or 0. We can calculate

$$
\begin{aligned}
\mathrm{E}\left[\left(y_i - \bar{y}\right)^2\right] =& \mathrm{E}\left[y_i^2 + \bar{y}^2 - 2 y_i \bar{y}\right] \\
=& \mathrm{E}\left[y_i + \bar{y}^2 - 2 y_i \bar{y}\right] \\
=& \mathrm{E}\left(y_i\right) + \mathrm{E}\left(\bar{y}^2\right) - 2\mathrm{E}\left(y_i \bar{y}\right)
\end{aligned}
$$

We have $\mathrm{E}\left(y_i\right)=\pi_i$. Based on Formula (A4), we also can calculate

$$\begin{aligned}
\mathrm{E}\left(y_i \bar{y}\right) = & \mathrm{E}\left(y_i \frac{1}{n} \sum_{k=1}^{n} y_k\right) \\
= & \frac{1}{n} \sum_{k=1}^{n} \mathrm{E}\left(y_i y_k\right) \\
= & \frac{1}{n}\left[\mathrm{E}\left(y_i^2\right)+\sum_{k=1, k \neq i}^{n} \mathrm{E}\left(y_i y_k\right)\right] \\
= & \frac{1}{n}\left[\mathrm{E}\left(y_i\right)+\sum_{k=1, k \neq i}^{n} \mathrm{E}\left(y_i\right) \mathrm{E}\left(y_k\right)\right] \\
= & \frac{1}{n}\left[\pi_i+\sum_{k=1, k \neq i}^{n} \pi_i \pi_k\right] \\
= & \frac{1}{n}\left[\pi_i+\pi_i \sum_{k=1, k \neq i}^{n} \pi_k\right] \\
= & \frac{1}{n}\left\{\pi_i+\pi_i\left[\sum_{k=1}^{n} \pi_k-\pi_i\right]\right\} \\
= & \frac{1}{n}\left\{\pi_i\left(1-\pi_i\right)+\pi_i \sum_{k=1}^{n} \pi_k\right\}
\end{aligned}$$

Hence, we can get

$$\begin{aligned}
\mathrm{E}\left(y_i-\bar{y}\right)^2 = & \pi_i+\frac{1}{n^2} \sum_{k=1}^{n} \pi_k\left(1-\pi_k\right)+\left[\frac{1}{n} \sum_{k=1}^{n} \pi_k\right]^2 \\
& -\frac{2}{n}\left[\pi_i\left(1-\pi_i\right)+\pi_i \sum_{k=1}^{n} \pi_k\right]
\end{aligned} \tag{A6}$$

We next calculate $\mathrm{E}\left(y_i-\bar{y}\right)\left(y_j-\bar{y}\right)$ for $i \neq j$:

$$\begin{aligned}
& \mathrm{E}\left(y_i-\bar{y}\right)\left(y_j-\bar{y}\right) \\
= & \mathrm{E}\left(y_i y_j-y_i \bar{y}-y_j \bar{y}+\bar{y}^2\right) \\
= & \mathrm{E}\left(y_i\right) \mathrm{E}\left(y_j\right)-\mathrm{E}\left(y_i \bar{y}\right)-\mathrm{E}\left(y_j \bar{y}\right)+\mathrm{E}\left(\bar{y}^2\right) \\
= & \pi_i \pi_j-\frac{1}{n}\left[\pi_i\left(1-\pi_i\right)+\pi_i \sum_{k=1}^n \pi_k\right]-\frac{1}{n}\left[\pi_j\left(1-\pi_j\right)+\pi_j \sum_{k=1}^n \pi_k\right] \\
& +\frac{1}{n^2} \sum_{k=1}^n \pi_k\left(1-\pi_k\right)+\left[\frac{1}{n} \sum_{k=1}^n \pi_k\right]^2
\end{aligned} \tag{A7}$$

Therefore, we can get

$$\begin{aligned}
\mathrm{E}\left(U_1 U_2\right)= & \sum_{i=1}^n x_i z_i\left\{\pi_i+\frac{1}{n^2} \sum_{k=1}^n \pi_k\left(1-\pi_k\right)+\left[\frac{1}{n} \sum_{k=1}^n \pi_k\right]^2\right. \\
& \left.-\frac{2}{n}\left[\pi_i\left(1-\pi_i\right)+\pi_i \sum_{k=1}^n \pi_k\right]\right\} \\
& +\sum_{i \neq j} x_i z_j\left\{\pi_i \pi_j-\frac{1}{n}\left[\pi_i\left(1-\pi_i\right)+\pi_i \sum_{k=1}^n \pi_k\right]\right. \\
& -\frac{1}{n}\left[\pi_j\left(1-\pi_j\right)+\pi_j \sum_{k=1}^n \pi_k\right] \\
& \left.+\frac{1}{n^2} \sum_{k=1}^n \pi_k\left(1-\pi_k\right)+\left[\frac{1}{n} \sum_{k=1}^n \pi_k\right]^2\right\}
\end{aligned} \tag{A8}$$

Therefore we then can get under H_0'

$$
\begin{aligned}
\widehat{\mathrm{E}\left[U_1 U_2\right]} \overset{H_0'}{=} & \sum_{i=1}^{n} x_i z_i \left\{ \bar{y} + \frac{1}{n^2} \sum_{k=1}^{n} \bar{y}\left(1-\bar{y}\right) + \left[\frac{1}{n} \sum_{k=1}^{n} \bar{y}\right]^2 \right. \\
& \left. - \frac{2}{n} \left[\bar{y}\left(1-\bar{y}\right) + \bar{y} \sum_{k=1}^{n} \bar{y}\right] \right\} \\
& + \sum_{i \neq j} x_i z_j \left\{ \bar{y}\bar{y} - \frac{1}{n}\left[\bar{y}\left(1-\bar{y}\right) + \bar{y} \sum_{k=1}^{n} \bar{y}\right] - \frac{1}{n}\left[\bar{y}\left(1-\bar{y}\right) + \bar{y} \sum_{k=1}^{n} \bar{y}\right] \right. \\
& \left. + \frac{1}{n^2} \sum_{k=1}^{n} \bar{y}\left(1-\bar{y}\right) + \left[\frac{1}{n} \sum_{k=1}^{n} \bar{y}\right]^2 \right\} \\
= & \sum_{i=1}^{n} x_i z_i \left\{ \bar{y} + \frac{1}{n}\bar{y}\left(1-\bar{y}\right) + \bar{y}^2 - \frac{2}{n}\bar{y}\left(1-\bar{y}\right) - 2\bar{y}^2 \right\} \\
& + \sum_{i \neq j} x_i z_j \left\{ \bar{y}^2 - \frac{1}{n}\bar{y}\left(1-\bar{y}\right) - \bar{y}^2 - \frac{1}{n}\bar{y}\left(1-\bar{y}\right) - \bar{y}^2 + \frac{1}{n}\bar{y}\left(1-\bar{y}\right) + \bar{y}^2 \right\} \\
= & \sum_{i=1}^{n} x_i z_i \left\{ \bar{y} - \bar{y}^2 - \frac{1}{n}\bar{y}\left(1-\bar{y}\right) \right\} - \frac{1}{n}\bar{y}\left(1-\bar{y}\right) \sum_{i \neq j} x_i z_j \\
= & \bar{y}\left(1-\bar{y}\right) \sum_{i=1}^{n} x_i z_i - \frac{1}{n}\bar{y}\left(1-\bar{y}\right) \left[\sum_{i=1}^{n} x_i z_i + \sum_{i \neq j} x_i z_j\right] \\
= & \bar{y}\left(1-\bar{y}\right) \sum_{i=1}^{n} x_i z_i - \frac{1}{n}\bar{y}\left(1-\bar{y}\right) \sum_{i=1}^{n} x_i \sum_{j=1}^{n} z_j \\
= & \bar{y}\left(1-\bar{y}\right) \left[\sum_{i=1}^{n} x_i z_i - \frac{1}{n} \sum_{i=1}^{n} x_i \sum_{j=1}^{n} z_j\right] \\
= & \bar{y}\left(1-\bar{y}\right) \left[\sum_{i=1}^{n} x_i z_i - n\bar{x}\bar{z}\right] \\
= & \bar{y}\left(1-\bar{y}\right) \left[\sum_{i=1}^{n} \left(x_i - \bar{x}\right)\left(z_i - \bar{z}\right)\right]
\end{aligned}
$$

Therefore, we have

$$\widehat{\text{Cov }(\mathbf{U})} \overset{H_0'}{=} \bar{y}\left(1-\bar{y}\right)\begin{pmatrix} \sum_{i=1}^{n}\left(x_i-\bar{x}\right)^2 & \sum_{i=1}^{n}\left(x_i-\bar{x}\right)\left(z_i-\bar{z}\right) \\ \sum_{i=1}^{n}\left(x_i-\bar{x}\right)\left(z_i-\bar{z}\right) & \sum_{j=1}^{n}\left(z_j-\bar{z}\right)^2 \end{pmatrix}$$
$$= n\bar{y}\left(1-\bar{y}\right)\begin{pmatrix} \hat{\sigma}_x^2 & \hat{\sigma}_{xz} \\ \hat{\sigma}_{xz} & \hat{\sigma}_z^2 \end{pmatrix},$$

where $\hat{\sigma}_x^2 = \frac{1}{n}\sum_{i=1}^{n}(x_i-\bar{x})^2$ and $\hat{\sigma}_z^2 = \frac{1}{n}\sum_{i=1}^{n}(z_i-\bar{z})^2/n$ are the sample variances for x_i and z_i respectively, and $\hat{\sigma}_{xz} = \frac{1}{n}\sum_{i=1}^{n}(x_i-\bar{x})(z_i-\bar{z})/n$ is the sample covariance between x_i and z_i.

Note that in logistic regression (A1), the random variables are y_i, while x_i and z_i are fixed (i.e., non-random). Hence, the (asymptotic) distributions of the U_1, U_2, and T do not depend on the distributions of x_i and z_i. In this sense, we can say that the joint statistic T are robust to the violation of the normality assumptions for the predictors x_i and z_i.

Based on Dobson (1990) [2],

$$\mathbf{U} \overset{H_0'}{\to} N(0, Cov(\mathbf{U})).$$

Denote $\mathbf{\Omega} = Cov(\mathbf{U}|H_0')$. We have

$$\mathbf{\Omega}^{-1/2}\mathbf{U} \overset{H_0'}{\to} N(0, \mathbf{I}_2).$$

By the relationship between multivariate normal distribution and chi square distribution, we have

$$\left(\mathbf{\Omega}^{-1/2}\mathbf{U}\right)^T\left(\mathbf{\Omega}^{-1/2}\mathbf{U}\right) = \mathbf{U}^T\mathbf{\Omega}^{-1}\mathbf{U} \overset{H_0'}{\to} \chi_2^2.$$

Based on the Law of Large Numbers, we have

$$\widehat{Cov(\mathbf{U})} \overset{H_0'}{\to} Cov(\mathbf{U}).$$

Hence, we have

$$T = \mathbf{U}^T \left[\widehat{Cov\left(\mathbf{U}\right)}\right]^{-1} \mathbf{U} \overset{H_0'}{\to} \chi_2^2. \tag{A9}$$

Note that we can derive an estimate of $Cov(\mathbf{U})$ under the alternative hypothesis based on formulas (A3), (A5), and (A8).

B Quality control and Data preprocessing

B.1 QC and preprocessing of HumanMethylation27 data sets

As the cleaning method in Li et.al (2015) [3], we removed the CpG sites with low quality (containing missing values or residing near SNP). The quantile plots has been presented in Li et.al (2015) [3] and did not show any obvious pattern. We did principle component analysis on the two data set. The scatter plot of the first principal component (PC1) versus the second principal component (PC2) for GSE37020 and GSE20080 did not show any obvious pattern (c.f. the left and right panels of Figure S1).

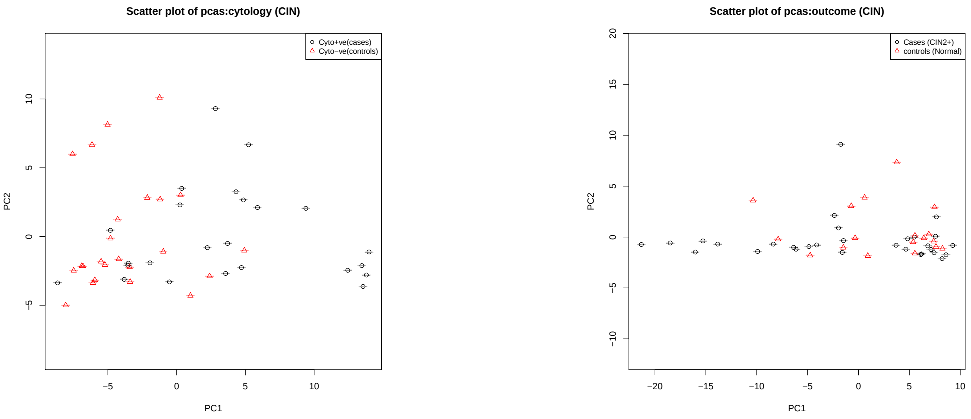

Figure S1: The plot of the first principal component (PC1) versus the second principal component (PC2) for HumanMethylation27 data. Left panel was for GSE37020; right panel was for GSE20080.

Next we obtained residual for each sample after regressing out the effect of age from methylation levels. We re-did principle component analysis on the adjusted data and did not find any obvious patterns (c.f. the left and right panels of Figure S2).

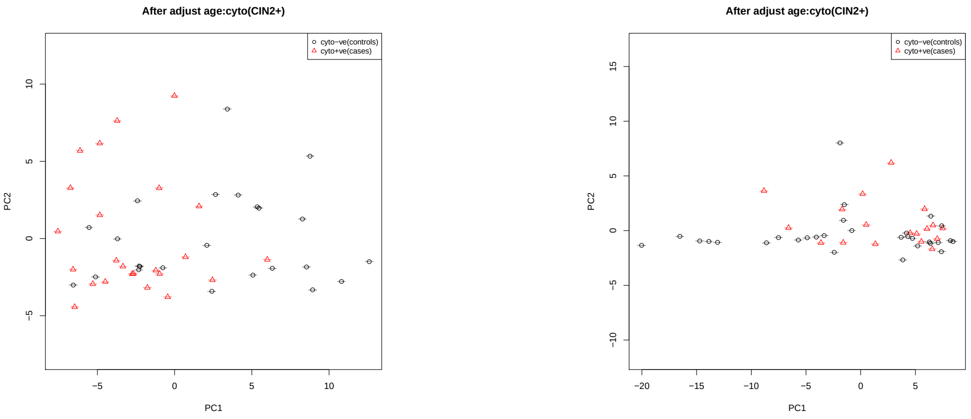

Figure S2: The plot of the first principal component (PC1) versus the second principal component (PC2) for HumanMethylation27 data after adjusting the effect of age. Left panel was for GSE37020; right panel was for GSE20080.

We also applied the 6 joint tests on the adjusted 27k data sets after regressing out the effect of age. Table S1 displayed that our three improved methods performed better than jointLRT and KS. And iAW.Lev and iAW.TM performed better than AW. This is consistent

with the results of unadjusted data (Table 5 in main text).

Table S1: The performances of 6 joint tests on adjusted Human-Methylation27k data GSE37020 and GSE20080.

Test	nSig	nValidation	nTV	pTV(%)	nFV	pFV(%)
JointLRT	3735	2044	1590	77.8	454	22.2
KS	190	31	26	83.9	5	16.1
AW	651	111	98	88.3	13	11.7
iAW.Lev	985	440	407	92.5	33	7.5
iAW.BF	824	176	155	88.1	21	11.9
iAW.TM	764	201	184	91.5	17	8.5

nSig : the number of significant CpG sites detected in GSE37020 based on FDR adjusted p-value < 0.05;
nValidation : the number of validated CpG sites in GSE20080 based on unadjusted p-value < 0.05;
nTV : the number of truly validated CpG sites with the same difference directions in means and variances between the two groups;
pTV : $= \frac{nTV}{nSigValid}$, the proportion of significant CpG sites detected in GSE37020 and truly validated in GSE20080;
nFV : the number of falsely validated CpG sites in GSE20080 with inconsistent difference direction in means or variances between the two groups;
pFV : $= \frac{nFV}{nSigValid}$, the proportion of significant CpG sites detected in GSE37020 but falsely validated in GSE20080.

B.2 QC and preprocessing of Illumina MethylationEPIC data

For the Illumina MethylationEPIC data set GSE107080, we initially removed the CpG sites with at least one missing value or with probe name using “ch” as prefix. Secondly, those CpG sites with detection p-values larger than or equal to $1e^{-12}$ are discarded. We calculated the quantiles across arrays and drew the plot of quantiles. No obvious patterns were found.

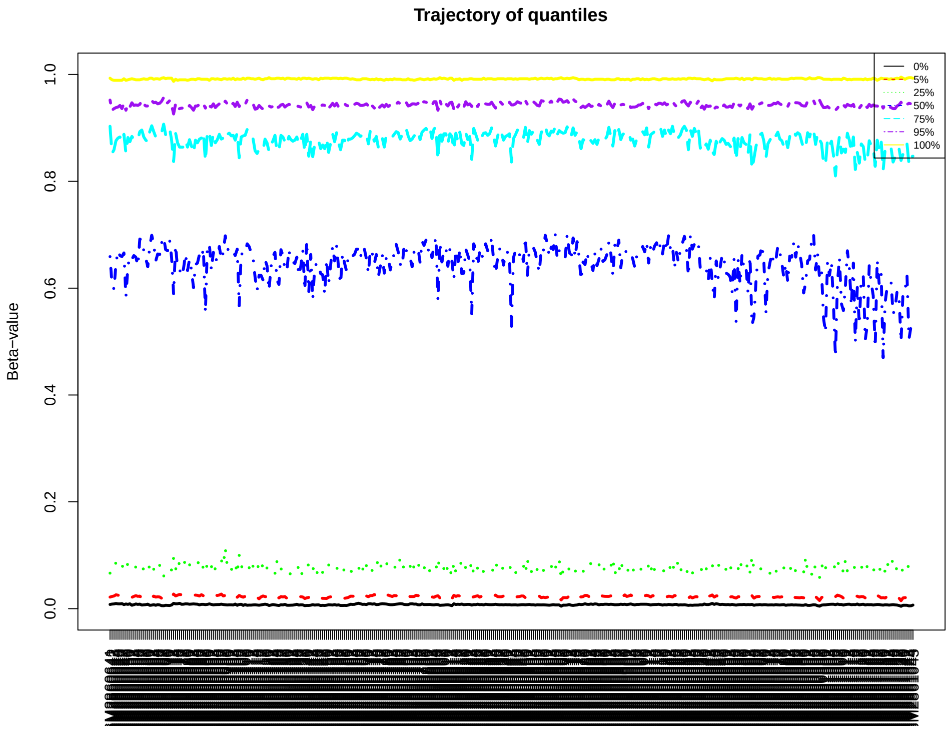

Figure S3: The plot of quantiles across arrays for GSE107080.

Then we did principal component analysis for the cleaned GSE107080 data set. The results did not show any obvious patterns (c.f. the left panel in Figure). Additionally, we regressed out the effects of age and cell type compositions and obtained the residuals. We re-did principle component analysis on the adjusted data and drew the plot of the first two principal components. No obvious patterns were found in the adjusted data (c.f. the left panel in Figure)

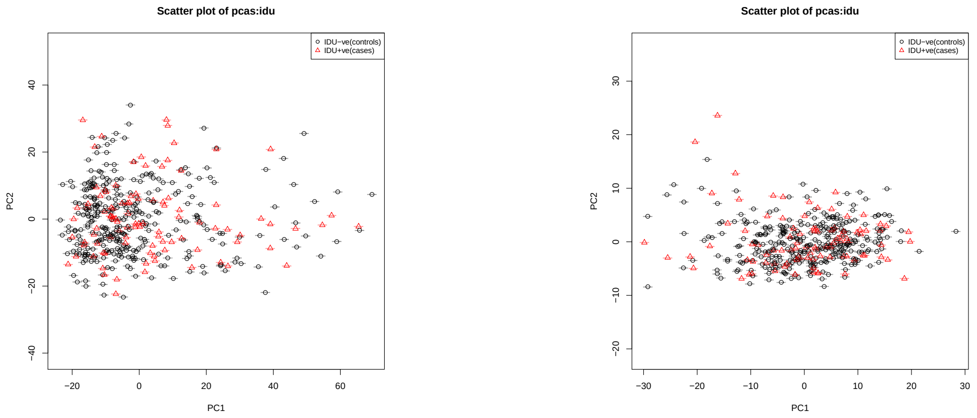

Figure S4: The plot of the first principal component (PC1) versus the second principal component (PC2) for MethylationEPIC data GSE107080. Left panel was for unadjusted data; right panel was for adjusted data.

C Additional simulation results

Table S2: The empirical Type I error rates (×100) and power (×100) for the six tests evaluated at 5%, 1% and 0.5% significance levels when methylation values were generated from normal distributions without or with an outlier. The numbers of non-diseased and diseased samples are (50,50).

Scenarios	Out	Level(%)	jointLRT	KS	AW	iAW.Lev	iAW.BF	iAW.TM
eqM&eqV	No	5	5.5	3.8	5.0	5.1	5.1	5.1
(Type I error)	No	1	1.1	0.5	0.8	0.9	0.9	0.9
	No	0.5	0.5	0.3	0.4	0.4	0.4	0.5
diffM&eqV	No	5	75.8	68.7	77	76.3	76.6	76.6
	No	1	53.1	40.8	53.5	52.9	53.2	53.6
	No	0.5	42.9	40.8	44.7	43.5	44.9	43.2
eqM&diffV	No	5	60.5	10.3	55.6	51.8	49.9	50.9
	No	1	35.5	2.0	25.9	27.7	26.1	27.6
	No	0.5	26.5	2.0	18.2	20.3	19.1	18.8
diffM&diffV	No	5	49.5	30	48.8	47.2	46.3	47.0
	No	1	27.1	10.0	25	23.6	23.2	23.9
	No	0.5	19.0	10.0	18.4	17.3	17.6	16.8
eqM&eqV	Yes	5	19.0	3.9	3.0	4.6	4.6	4.6
(Type I error)	Yes	1	6.4	0.5	0.4	0.7	0.7	0.7
	Yes	0.5	4.0	0.3	0.2	0.2	0.3	0.3
diffM&eqV	Yes	5	64.4	72.1	84.4	81.8	82.1	81.8
	Yes	1	33.9	44.3	63.9	59.7	59.5	60.0
	Yes	0.5	24.3	34.9	56.7	52.2	51.5	50.0
eqM&diffV	Yes	5	6.3	9.1	19.7	29.7	29.2	30.0
	Yes	1	1.1	2.0	7.0	12.3	11.8	12.6
	Yes	0.5	0.6	1.0	4.9	9.2	8.5	8.6
diffM&diffV	Yes	5	13.9	32.6	45.8	46.1	47.2	47.3
	Yes	1	3.4	11.0	23.7	23.2	24.1	24.5
	Yes	0.5	1.7	7.3	18.9	18.3	19.0	18.2

Table S3: The empirical Type I error rates (×100) and power (×100) for the six tests evaluated at 5%, 1% and 0.5% significance levels when methylation values were generated from Beta distributions without or with an outlier. The numbers of non-diseased and diseased samples are (50,50).

Scenarios	Out	Level(%)	jointLRT	KS	AW	iAW.Lev	iAW.BF	iAW.TM
eqM&eqV	No	5	6.0	3.6	5.4	5.5	5.3	5.5
(Type I error)	No	1	1.3	0.4	1.0	1.2	1.2	1.2
	No	0.5	0.6	0.2	0.5	0.6	0.6	0.6
diffM&eqV	No	5	73.7	70.2	77.3	75.3	76.8	76.3
	No	1	50.8	41.6	54.1	50.6	51.7	50.8
	No	0.5	42.1	41.6	46.0	40.9	43.0	42.2
eqM&diffV	No	5	56.7	9.9	52.7	49.8	49.1	49.8
	No	1	31.2	1.5	24.0	23.9	21.9	22.6
	No	0.5	23.8	1.5	16.7	16.7	15.7	16.5
diffM&diffV	No	5	50.5	35.2	56.8	52.2	53.7	53.8
	No	1	24.6	13.1	31.2	25.7	26.8	26.8
	No	0.5	17.2	13.1	23.4	17.8	20.0	19.8
eqM&eqV	Yes	5	16.1	3.8	3.3	4.7	4.5	4.7
(Type I error)	Yes	1	4.3	0.5	0.5	0.8	0.8	0.8
	Yes	0.5	2.4	0.2	0.3	0.4	0.4	0.4
diffM&eqV	Yes	5	73.8	74.2	85.5	84.1	84.9	84.2
	Yes	1	48.7	46.1	65.1	61.3	62.4	61.4
	Yes	0.5	37.6	46.1	55.6	51.5	50.9	51.1
eqM&diffV	Yes	5	2.5	8.4	6.4	19.5	18.7	18.7
	Yes	1	0.5	1.2	1.7	6.7	6.2	6.3
	Yes	0.5	0.2	1.2	0.9	4.1	3.5	3.7
diffM&diffV	Yes	5	2.8	28.4	13.4	10.8	10.9	10.7
	Yes	1	0.7	9.7	4.8	2.4	2.5	2.4
	Yes	0.5	0.4	9.7	3.2	1.4	1.4	1.4

Table S4: The empirical Type I error rates ($\times 100$) and power ($\times 100$) for the six tests evaluated at 5%, 1% and 0.5% significance levels when methylation values were generated from mixtures of two normal distributions without or with an outlier. The numbers of non-diseased and diseased samples are (50,50).

Scenarios	Out	Level(%)	jointLRT	KS	AW	iAW.Lev	iAW.BF	iAW.TM
eqM&eqV	No	5	2.4	4	4.5	8.9	5.3	10.0
(Type I error)	No	1	0.5	0.6	0.9	2.7	1.4	2.8
	No	0.5	0.3	0.3	0.4	1.6	0.7	1.5
diffM&eqV	No	5	9.8	29.8	44.2	26.5	49.9	27.7
	No	1	2.2	10.5	20.4	7.9	23.2	10.0
	No	0.5	1.1	7.0	14.8	5.0	15.0	6.3
eqM&diffV	No	5	18.5	68.3	36.4	59	35.3	46
	No	1	3.6	31.5	19.7	33.3	16.7	26.5
	No	0.5	1.7	22.1	15.7	27.1	11.3	20.3
diffM&diffV	No	5	21.2	71.9	41.1	63.6	41.6	51.2
	No	1	4.8	34.4	23.6	37.7	21	30.7
	No	0.5	2.3	25.6	18.3	30.3	14.9	24.6
eqM&eqV	Yes	5	47.0	3.9	2.2	4.4	4.2	5.0
(Type I error)	Yes	1	15.2	0.6	0.3	0.8	0.8	0.9
	Yes	0.5	9.2	0.3	0.1	0.3	0.4	0.4
diffM&eqV	Yes	5	3.2	31.4	11.3	10.2	39.5	15.3
	Yes	1	0.5	11.2	3.2	2.6	19.8	3.9
	Yes	0.5	0.3	7.4	1.8	1.7	13.0	2.5
eqM&diffV	Yes	5	0.2	66.1	7.4	39.6	24.7	33.1
	Yes	1	0.1	30.6	2.2	19.2	11.6	15.1
	Yes	0.5	0.0	21.8	1.3	14.8	7.7	11.5
diffM&diffV	Yes	5	0.3	69.8	10.6	44.9	32.8	39.4
	Yes	1	0.0	34.6	3.8	24.3	16.4	20.2
	Yes	0.5	0.0	25.2	2.4	19.2	11.0	15.3

Table S5: The empirical Type I error rates (×100) and power (×100) for the six tests evaluated at 5%, 1% and 0.5% significance levels when methylation values were generated from Chi-square distributions without or with an outlier. The numbers of non-diseased and diseased samples are (50,50).

Scenarios	Out	Level(%)	jointLRT	KS	AW	iAW.Lev	iAW.BF	iAW.TM
eqM&eqV	No	5	13.8	3.7	4.8	6.1	5.1	5.1
(Type I error)	No	1	5.9	0.5	0.7	1.3	1.0	0.9
	No	0.5	4.3	0.2	0.4	0.8	0.5	0.5
diffM&eqV	No	5	50.1	88.1	91.8	85.7	94.2	93.6
	No	1	21.3	64.9	78.9	63.4	82.7	81.5
	No	0.5	12.8	64.9	71.6	50.5	75.5	72.2
eqM&diffV	No	5	12.0	6.9	15.9	16.3	18.5	18.3
	No	1	3.4	0.9	5.2	5.3	6.2	6.3
	No	0.5	2.0	0.9	3.0	3.0	3.9	3.6
diffM&diffV	No	5	11.4	21.7	34.5	28.0	39.7	38.7
	No	1	3.0	6.1	16.1	10.5	19	18.2
	No	0.5	1.5	6.1	10.6	6.2	13.4	11.7
eqM&eqV	Yes	5	23.1	3.6	3.3	6.4	5.0	5.0
(Type I error)	Yes	1	12.2	0.5	0.4	1.4	0.8	0.8
	Yes	0.5	9.3	0.2	0.2	0.6	0.4	0.3
diffM&eqV	Yes	5	16.4	85.2	93.3	82.5	93.1	92.6
	Yes	1	2.6	59.6	82.7	57.1	81.5	79.8
	Yes	0.5	1.6	50.1	76.8	47.3	75.5	72.8
eqM&diffV	Yes	5	19.8	6.3	19.4	24.7	24.0	23.8
	Yes	1	5.6	0.7	4.4	7.9	7.9	7.5
	Yes	0.5	3.6	0.4	2.5	4.6	4.9	5.0
diffM&diffV	Yes	5	14.2	18.8	39.9	34.3	43.5	42.3
	Yes	1	3.9	4.8	18.0	13.4	22.3	21.1
	Yes	0.5	2.2	2.8	12.6	8.9	16.3	14.9

Table S6: The empirical Type I error rates (×100) and power (×100) for the six tests evaluated at 5%, 1% and 0.5% significance levels when methylation values were generated from normal distributions without or with an outlier. The numbers of non-diseased and diseased samples are (20,20).

Scenarios	Out	Level(%)	jointLRT	KS	AW	iAW.Lev	iAW.BF	iAW.TM
eqM&eqV	No	5	5.9	3.3	5.3	5.4	5.7	5.8
(Type I error)	No	1	1.4	0.4	0.9	0.9	1.2	1.0
	No	0.5	0.8	0.4	0.4	0.4	0.5	0.4
diffM&eqV	No	5	35.5	29.2	35.2	33.7	34.1	33.6
	No	1	15.2	9.1	16.0	14.7	14.5	14.6
	No	0.5	10.9	9.1	11.8	9.7	9.8	10.0
eqM&diffV	No	5	25.6	4.8	19.8	19.9	17.9	18.5
	No	1	8.4	0.7	4.6	6.2	4.6	5.8
	No	0.5	5.4	0.7	2.6	3.4	2.6	3.2
diffM&diffV	No	5	22.3	11.7	20.6	20.1	18.5	19.1
	No	1	6.7	2.7	6.5	6.4	5.5	6.2
	No	0.5	4.2	2.7	4.3	3.8	3.7	4.0
eqM&eqV	Yes	5	26.2	3.1	2.6	3.5	3.8	3.7
(Type I error)	Yes	1	10.6	0.4	0.3	0.3	0.5	0.5
	Yes	0.5	7.2	0.4	0.1	0.1	0.2	0.2
diffM&eqV	Yes	5	22.5	34.7	45.7	42.4	42.9	42.4
	Yes	1	6.4	11.7	21.1	19.3	19.2	19.0
	Yes	0.5	3.2	11.7	15.3	15.1	12.2	12.6
eqM&diffV	Yes	5	0.6	4.2	8.2	9.3	9.9	10.4
	Yes	1	0.1	0.5	2.1	2.9	2.9	3.1
	Yes	0.5	0.1	0.5	1.3	2.1	1.8	1.9
diffM&diffV	Yes	5	3.5	13.9	23.3	21.1	22.8	22.7
	Yes	1	0.5	3.6	8.3	7.9	8.4	8.6
	Yes	0.5	0.2	3.6	5.6	5.8	5.0	5.3

Table S7: The empirical Type I error rates (×100) and power (×100) for the six tests evaluated at 5%, 1% and 0.5% significance levels when methylation values were generated from Beta distributions without or with an outlier. The numbers of non-diseased and diseased samples are (20,20).

Scenarios	Out	Level(%)	jointLRT	KS	AW	iAW.Lev	iAW.BF	iAW.TM
eqM&eqV	No	5	6.2	3.3	5.4	5.5	5.5	5.7
(Type I error)	No	1	1.5	0.4	0.9	0.8	1.0	1.0
	No	0.5	0.8	0.4	0.3	0.3	0.5	0.4
diffM&eqV	No	5	36	30.8	37.4	35.2	36.3	35.4
	No	1	14.8	9.7	17.8	16.8	16.7	16.7
	No	0.5	10.1	9.7	13.4	12.1	11.1	11.5
eqM&diffV	No	5	22.9	4.7	18.0	18.9	16.8	17.6
	No	1	7.2	0.4	4.9	6.3	4.6	5.3
	No	0.5	4.7	0.4	2.6	3.9	2.6	2.9
diffM&diffV	No	5	20.9	14.0	23.0	21.2	21.3	21.2
	No	1	6.3	3.2	8.2	8.1	8.1	8.1
	No	0.5	3.7	3.2	5.5	5.2	4.9	5.3
eqM&eqV	Yes	5	23.5	3.3	2.6	3.6	4.1	4.0
(Type I error)	Yes	1	6.4	0.4	0.2	0.3	0.5	0.5
	Yes	0.5	3.4	0.4	0.1	0.1	0.2	0.2
diffM&eqV	Yes	5	32.4	36.3	46.5	45.0	43.7	43.3
	Yes	1	13.7	12.1	22.4	20.1	19.1	19.3
	Yes	0.5	7.8	12.1	16.2	13.7	13.1	13.5
eqM&diffV	Yes	5	0.3	4.2	3.8	4.7	5.4	5.2
	Yes	1	0.0	0.3	1.1	1.3	1.6	1.7
	Yes	0.5	0.0	0.3	0.5	0.8	1.1	1.1
diffM&diffV	Yes	5	1.6	9.3	8.4	3.4	3.7	3.6
	Yes	1	0.3	1.8	3.1	0.9	1.2	1.1
	Yes	0.5	0.1	1.8	2.1	0.5	0.6	0.6

Table S8: The empirical Type I error rates (×100) and power (×100) for the six tests evaluated at 5%, 1% and 0.5% significance levels when methylation values were generated from mixtures of two normal distributions without or with an outlier. The numbers of non-diseased and diseased samples are (20,20).

Scenarios	Out	Level(%)	jointLRT	KS	AW	iAW.Lev	iAW.BF	iAW.TM
eqM&eqV	No	5	3.4	3.3	4.5	8.1	5.1	6.7
(Type I error)	No	1	0.8	0.5	0.8	2.4	1.3	1.6
	No	0.5	0.5	0.5	0.4	1.3	0.6	0.9
diffM&eqV	No	5	5.8	11	17.7	10.8	17.5	12.6
	No	1	1.3	2	5.2	2.0	4.9	2.8
	No	0.5	0.7	2	3.1	0.9	2.4	1.1
eqM&diffV	No	5	11.2	24.4	26.2	28.5	23.1	27.3
	No	1	2.1	5.5	13.8	11.2	10	12.2
	No	0.5	0.9	5.5	10.2	6.3	6.9	7.5
diffM&diffV	No	5	11.8	25.4	29.1	31.1	25.9	30.4
	No	1	1.9	6.8	15.8	11.9	10.8	13.3
	No	0.5	0.8	6.8	11.5	6.2	7.2	8.1
eqM&eqV	Yes	5	60.5	3.4	1.9	2.9	2.2	2.3
(Type I error)	Yes	1	23.6	0.5	0.2	0.5	0.3	0.3
	Yes	0.5	14.2	0.5	0.1	0.2	0.1	0.1
diffM&eqV	Yes	5	2.7	12.7	9.0	7.0	17.3	10.4
	Yes	1	0.7	2.8	2.2	2.3	6.9	3.4
	Yes	0.5	0.2	2.8	1.2	1.2	4.0	2.0
eqM&diffV	Yes	5	0.3	22.8	8.4	13.6	16.0	16.4
	Yes	1	0.0	5.6	2.3	5.6	7.3	7.4
	Yes	0.5	0.0	5.6	1.6	3.5	5.0	5.1
diffM&diffV	Yes	5	0.7	26	10.4	16.3	19.7	20.3
	Yes	1	0.1	7.2	3.8	6.6	9.7	9.6
	Yes	0.5	0.1	7.2	2.6	4.2	6.9	6.7

Table S9: The empirical Type I error rates (×100) and power (×100) for the six tests evaluated at 5%, 1% and 0.5% significance levels when methylation values were generated from Chi-square distributions without or with an outlier. The numbers of non-diseased and diseased samples are (20,20).

Scenarios	Out	Level(%)	jointLRT	KS	AW	iAW.Lev	iAW.BF	iAW.TM
eqM&eqV	No	5	13.4	3.2	5.3	6.4	5.3	5.5
(Type I error)	No	1	5.2	0.4	0.9	1.3	1.0	0.9
	No	0.5	3.7	0.4	0.4	0.6	0.5	0.5
diffM&eqV	No	5	23.9	45.3	52.4	41.0	55.7	52.8
	No	1	7.4	17.0	26.6	17.5	30.4	28.3
	No	0.5	4.6	17	19.4	10.6	22.6	19.2
eqM&diffV	No	5	7.7	3.9	8.7	8.8	9.2	9.2
	No	1	2.0	0.6	1.7	2.2	2.2	2.4
	No	0.5	1.2	0.6	1.0	1.1	1.1	1.2
diffM&diffV	No	5	8.5	9.8	15.5	13.0	18.5	16.9
	No	1	2.0	1.6	4.3	3.5	6.2	5.7
	No	0.5	1.1	1.6	2.5	1.5	3.7	2.7
eqM&eqV	Yes	5	29.7	3.0	2.7	5.9	4.0	3.8
(Type I error)	Yes	1	16.1	0.4	0.3	0.7	0.7	0.5
	Yes	0.5	12.3	0.4	0.1	0.2	0.3	0.2
diffM&eqV	Yes	5	4.0	35.2	55.9	36.1	52.9	52.3
	Yes	1	0.8	11.2	30.9	17.8	29.3	29.8
	Yes	0.5	0.2	11.2	23.8	12.1	20.6	22.1
eqM&diffV	Yes	5	11.9	3.6	9.1	13.3	11	12.2
	Yes	1	3.6	0.4	2.1	3.9	2.3	2.9
	Yes	0.5	2.0	0.4	1.0	2.1	1.0	1.3
diffM&diffV	Yes	5	9.2	6.8	18.2	17.0	20.3	20.7
	Yes	1	2.5	0.9	5.4	5.5	6.7	7.2
	Yes	0.5	1.3	0.9	3.4	3.3	3.7	4.0

References

[1] Ahn, S., Wang, T.: A powerful statistical method for indentifying differentially methylated markers in complex diseases. Pacific Symposium on Biocomputing, 69–79 (2013)

[2] Dobson, A.J.: An Introduction to Generalized Linear Models. Chapman and Hall (1990)

[3] Li, X., Qiu, W., Morrow, J., DeMeo, D.L., Weiss, S.T., Fu, Y., Wang, X.: A comparative study of tests for homogeneity of variances with application to dna methylation data. PloS one **10**(12), 0145295 (2015)
